# DNA methylation signatures for 2016 WHO classification subtypes of diffuse gliomas

**DOI:** 10.1186/s13148-017-0331-9

**Published:** 2017-04-04

**Authors:** Yashna Paul, Baisakhi Mondal, Vikas Patil, Kumaravel Somasundaram

**Affiliations:** Department of Microbiology and Cell Biology, Indian Institute of Science, Bangalore, 560012 India

**Keywords:** Glioma, DNA methylation classification signature, IDH1/IDH2 mutation, 2016 WHO, PAM, PCA

## Abstract

**Background:**

Glioma is the most common of all primary brain tumors with poor prognosis and high mortality. The 2016 World Health Organization classification of the tumors of central nervous system uses molecular parameters in addition to histology to redefine many tumor entities. The new classification scheme divides diffuse gliomas into low-grade glioma (LGG) and glioblastoma (GBM) as per histology. LGGs are further divided into isocitrate dehydrogenase (IDH) wild type or mutant, which is further classified into either oligodendroglioma that harbors 1p/19q codeletion or diffuse astrocytoma that has an intact 1p/19q loci but enriched for ATRX loss and TP53 mutation. GBMs are divided into IDH wild type that corresponds to primary or de novo GBMs and IDH mutant that corresponds to secondary or progressive GBMs. To make the 2016 WHO subtypes of diffuse gliomas more robust, we carried out Prediction Analysis of Microarrays (PAM) to develop DNA methylation signatures for these subtypes.

**Results:**

In this study, we applied PAM on a training set of diffuse gliomas derived from The Cancer Genome Atlas (TCGA) and identified DNA methylation signatures to classify LGG IDH wild type from LGG IDH mutant, LGG IDH mutant with 1p/19q codeletion from LGG IDH mutant with intact 1p/19q loci and GBM IDH wild type from GBM IDH mutant with an accuracy of 99–100%. The signatures were validated using the test set of diffuse glioma samples derived from TCGA with an accuracy of 96 to 99%. In addition, we also carried out additional validation of all three signatures using independent LGG and GBM cohorts. Further, the methylation signatures identified a fraction of samples as discordant, which were found to have molecular and clinical features typical of the subtype as identified by methylation signatures.

**Conclusions:**

Thus, we identified methylation signatures that classified different subtypes of diffuse glioma accurately and propose that these signatures could complement 2016 WHO classification scheme of diffuse glioma.

**Electronic supplementary material:**

The online version of this article (doi:10.1186/s13148-017-0331-9) contains supplementary material, which is available to authorized users.

## Background

The neoplasia of non-neuronal glial cells in the brain is referred to as glioma and is the most common type of primary central nervous system (CNS) tumors [1]. The different histological subtypes of glioma are as follows: astrocytoma being the most common, accounting for 70% of all cases, while oligodendroglioma comprises 9% which includes classic oligodendrogliomas as well as mixed oligoastrocytomas and ependymoma comprises 6% [2].

Over the past decades, classification of brain tumors was based on the histopathological and microscopic features in hematoxylin- and eosin-stained sections, like cell type, level of differentiation, identifying necrotic lesions, and presence of lineage-specific markers. According to the WHO 2007-based classification, grade II/diffused astrocytoma (DA) was described as low grade while high-grade glioma comprised of grade III/anaplastic astrocytoma (AA) and grade IV/glioblastoma (GBM) [3]. The vast majority of GBM develop de novo in elderly patients with no prior clinical or histological evidence and are referred to as primary GBM. Secondary GBM progresses through low-grade diffuse astrocytoma or anaplastic astrocytoma and is manifested in younger patients. Several studies have shown that glioma is highly heterogeneous which indicates that tumors of same grade have diverse genetic and epigenetic molecular aberrations [4–9]. With the invent of new technologies, many high-throughput studies have reported different molecular signatures based on glioma CpG island methylator phenotype (GCIMP), expression-based studies for mRNA, miRNA, and lncRNA in GBM [10–13]. One of the most exciting and clinically relevant observations was the discovery that a high percentage of grade II/III and grade IV secondary glioblastoma harbor mutations in the genes isocitrate dehydrogenase 1 and 2 [2]. Growing data indicate that these mutations play a causal role in gliomagenesis, have a major impact on tumor biology, and also have clinical and prognostic importance [2].

Nearly 12% of GBM patients have been identified to have point mutation in codon 132 (R132H) of the isocitrate dehydrogenase 1 (IDH1) gene located in the chromosome locus 2q33 [14]. IDH1 codes for a cytosolic protein that controls oxidative cellular damage [14, 15]. Several studies showed that the IDH1 mutation is inversely associated with grade in diffuse glial tumors, affecting 71% of grade II, 64% of grade III, and 6% of primary glioblastomas [14]. Interestingly, IDH mutation is found to be present in the secondary glioblastoma (76%) probably because these tumors have been derived from the lower grade gliomas [16]. IDH1 is an enzyme and it catalyzes the oxidative decarboxylation of isocitrate to produce α-ketoglutarate (α-KG) [17].

IDH mutation has been shown to be associated with alterations in the methylome thus being sufficient to establish glioma hypermethylator phenotype [18]. At present, 2016 WHO CNS tumor classification has included both molecular markers along with histological features to identify and classify different subtypes of diffuse glioma which includes the WHO grade II and grade III astrocytic tumors, the grade II and III oligodendrogliomas, and the grade IV glioblastomas. The low-grade gliomas (LGGs), which include the WHO grade II and grade III astrocytic tumors and the grade II and III oligodendrogliomas, are classified based on IDH mutation status. The LGG IDH mutant subtype is further classified based on the codeletion of 1p/19q where LGG IDH mutant patients harboring 1p/19q codeletion is termed as oligodendrogliomas (ODG) while LGG IDH mutant patients having intact 1p/19q loci are termed as diffuse astrocytoma which may be enriched in TP53 mutation/ATRX loss. The other axis is the glioblastoma (GBM) which, similar to LGG, is further classified into IDH WT and mutant. The deficiency in this classification is that factors like intra-tumoral heterogeneity and insufficient molecular information could result in our ability to classify certain samples to any specific categories. In such cases, signatures based on whole tumor studies to classify the glioma subtypes might further complement 2016 WHO classification.

In the present study, we investigated the altered methylation pattern among the different subtypes of diffuse gliomas as per 2016 WHO CNS tumor classification [19] and derived methylation-based classification signature for distinguishing different subtypes. Our study sets up the premise of using methylation signature in combination to the 2016 WHO classification system with a higher precision of classification of the diffuse glioma patients, thereby helping better diagnosis and appropriate treatment therapy.

## Result

### The overall work flow of methylation-based signatures to distinguish diffuse glioma subtypes of 2016 WHO classification

**Fig. 1:**
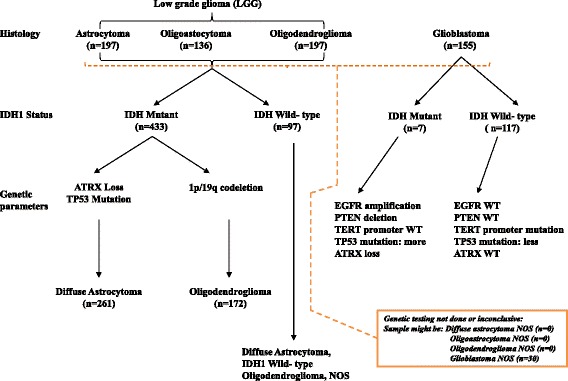
Overview of the 2016 WHO CNS tumor classification-based algorithm with the number of patients from TCGA dataset that is used in the present study

**Fig. 2:**
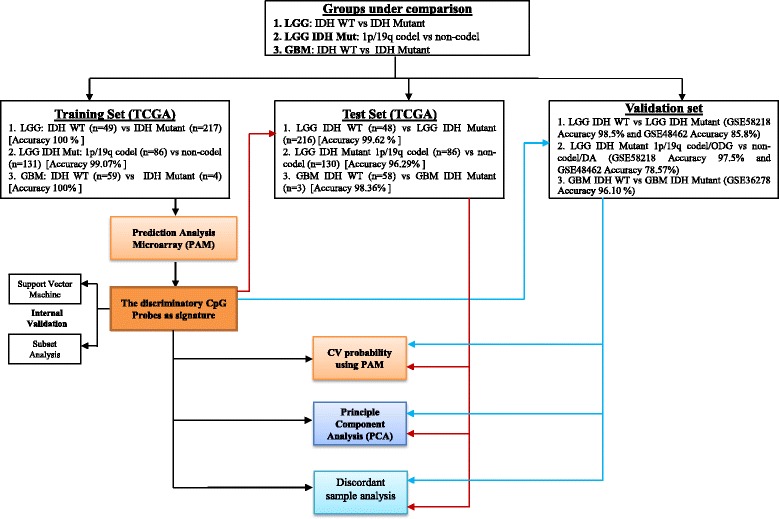
The schematic representation of the work flow of statistical analysis. PAM identified 14 discriminatory CpG probes of DNA methylation between (*1*) IDH Mut (LGG IDH Mut) and WT (LGG IDH WT) which was further validated by principal component analysis (PCA). Fourteen CpG probe methylation signatures were then validated in test set. Here, TCGA dataset (450K methylation) was randomly divided into equal halves to form the training and test set. Similar protocol was performed for (*2*) LGG IDH Mut 1p/19q intact (diffuse astrocytoma/DA) versus LGG IDH Mut 1p/19q codel (oligodendroglioma/ODG) and (*3*) GBM IDH Mut versus WT. All the derived methylation signatures are validated in independent validation datasets with high accuracy

To develop methylation-based signatures to distinguish diffuse glioma subtypes as per 2016 WHO CNS tumor classification (Fig. 1), we subjected the 450K DNA methylation data of The Cancer Genome Atlas (TCGA) diffuse glioma samples (https://cancergenome.nih.gov/) to various statistical tools and validation steps (Fig. 2). The methylation signatures were developed to distinguish LGG IDH mutant from LGG IDH WT, LGG IDH mutant with 1p/19q codeletion (oligodendroglioma) from LGG IDH mutant with intact 1p/19q loci (diffuse astrocytoma) and GBM IDH mutant (progressive GBM) from GBM IDH WT (de novo GBM). The TCGA samples were classified into these groups as per 2016 WHO classification scheme (Fig. 1). For methylation signature development, to begin with, we performed a Wilcoxon-rank sum test between different diffuse glioma subtypes to identify a list of significantly differentially methylated CpG probes, which were further subjected to a differential *β* value (Δ*β*) of 0.4 between groups. The TCGA samples were then divided randomly into two equal groups as training and test sets (Additional file 1: Table S1). The training set was subjected to Prediction Analysis of Microarrays (PAM) [20] to identify the methylation signatures containing minimum number of CpGs with least error. The robustness of the identified signatures was internally cross validated within training set using Support Vector Machine (SVM) [21] and subset validation. The signatures were further applied on the test set for the additional validation. Further, the signatures were subjected to external validation by using independent cohorts. We also used principal component analysis (PCA) to test the ability of methylation signatures to separate the two compared groups into two distinct clusters. Additionally, 10-fold cross-validation by PAM was carried out to identify the discordant samples, which were then subjected to further analysis to find out the true nature of these samples.

### 14 CpG methylation signatures to distinguish LGG IDH mutant from LGG IDH wild type (WT): identification and validation

**Fig. 3:**
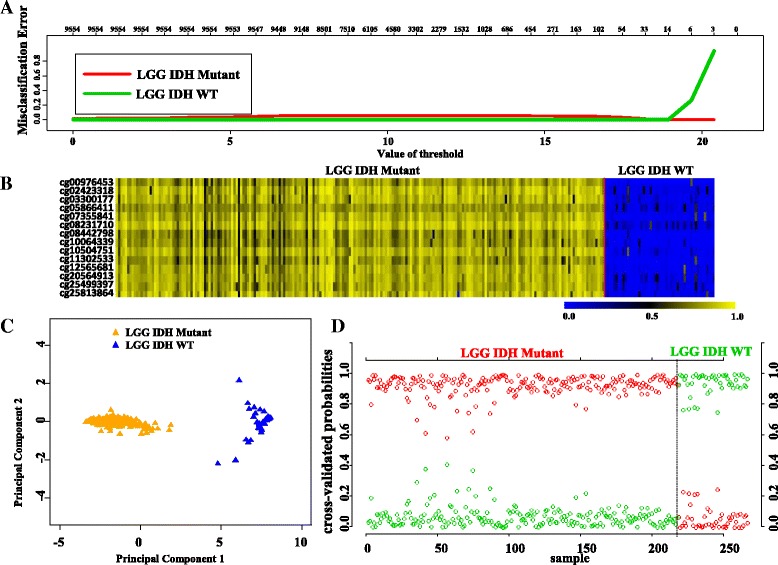
Identification of 14 CpG probe methylation signatures of LGG IDH mutant versus WT in training set (TCGA). **a** Plot demonstrating classification error for 9554 CpG probes from PAM analysis in training set. The threshold value 18.9 corresponded to 14 discriminatory CpG probes which classified IDH mutant (*n* = 217) and WT (*n* = 49) LGG samples with classification error of 0%. **b** Heat map of the 14 CpG discriminatory probes identified from the PAM analysis between LGG IDH Mut and WT patient samples in the training set (TCGA). A *dual color code* was used where *yellow* indicates more methylation (hypermethylation) and *blue* indicates less methylation (hypomethylation). **c** PCA was performed using beta (methylation) values of 14 PAM-identified CpG probes between IDH mutant (*n* = 217) and WT (*n* = 49) LGG samples in training set. A scatter plot is generated using the first two principal components for each sample. The *color code* of the samples is as indicated. **d** The detailed cross-validation probabilities of 10-fold cross-validation for the samples of training set based on the beta values of 14 CpG probes are shown. For each sample, its probability as LGG IDH Mut (*red color*) and WT (*green color*) is shown and it was predicted by the PAM program as either IDH Mut or WT in LGG samples based on which grade’s probability is higher. The original histological grade of the samples is shown on the *top*

PAM analysis of differentially methylated CpGs (Additional file 1: Table S2) in the training (TCAG) set (Additional file 1: Table S1) identified a set of 14 CpGs to distinguish IDH mutant from IDH WT in LGG at a threshold value of 18.9 with least error (Fig. 3a, Additional file 2: Figure S1A). The robustness of this probe set was tested by internal cross-validation using SVM, which gave a classification accuracy of 100% and subset validation with an accuracy of 100% (Additional file 2: Figure S2A and B respectively; see the Methods section for more details). The CpG probes of the signature were found to be hypermethylated in IDH mutant LGGs compared to IDH WT LGGs (Fig. 3b and Table 1). Further, upon subjecting the 14 CpG probes to PCA, the two principal components were able to form two distinct clusters for IDH mutant and IDH WT LGGs (Fig. 3c). Prediction accuracy estimation by 10-fold cross-validation using PAM showed that the 14 CpG probe methylation signatures predicted all LGG IDH mutant samples accurately with no error (Fig. 3d). Similarly, all LGG IDH WT samples were rightly predicted to be LGG with WT IDH samples based on the 14 CpG probe methylation signatures (Fig. 3d). Thus, the 14 CpG DNA methylation signatures were able to discriminate LGG IDH mutant from LGG IDH WT with an overall classification accuracy of 100%. The sensitivity and specificity of the signature for IDH mutant and WT in LGG are 100% (Table 2).

**Table 1:** List of the 14 CpG methylation signatures for LGG IDH mutant versus IDH WT in the training set and validation set (GSE58218)

			Training set (TCGA cohort)					Validation set (GSE58218 cohort)				
No.	CpG ID	Gene name	Average *β* in mutant	Average *β* in WT	∆*β* = (avg *β* in mutant−avg *β* in WT)	*p* value	FDR	Average *β* in mutant	Average *β* in WT	∆*β* = (avg *β* in mutant−avg *β* in WT)	*p* value	FDR
1	cg00976453	KCNB1	0.795	0.037	0.758	1.31E−27	1.67E−27	0.806	0.162	0.644	1.18E−19	1.38E−19
2	cg02423318	NA	0.860	0.096	0.764	9.56E−28	1.67E−27	0.876	0.167	0.709	7.35E−21	2.87E−20
3	cg03300177	GNAO1	0.841	0.063	0.777	9.78E−28	1.67E−27	0.839	0.171	0.667	1.47E−20	2.95E−20
4	cg05866411	FGFRL1	0.784	0.102	0.682	8.35E−28	1.67E−27	0.681	0.208	0.473	3.96E−20	5.54E−20
5	cg07355841	TPPP3	0.835	0.055	0.781	1.25E−27	1.67E−27	0.819	0.184	0.635	1.93E−20	3.38E−20
6	cg08231710	MMP23A	0.874	0.127	0.747	9.35E−28	1.67E−27	0.809	0.293	0.516	1.47E−18	1.58E−18
7	cg08442798	NA	0.772	0.023	0.749	8.35E−28	1.67E−27	0.824	0.090	0.734	5.43E−21	2.87E−20
8	cg10064339	UCP2	0.779	0.042	0.737	8.35E−28	1.67E−27	0.805	0.115	0.690	8.82E−21	2.87E−20
9	cg10504751	GNAO1	0.846	0.067	0.779	1.23E−27	1.67E−27	0.846	0.173	0.673	2.61E−20	4.06E−20
10	cg11302533	NA	0.784	0.037	0.747	1.8E−27	1.94E−27	0.834	0.102	0.732	1.19E−20	2.87E−20
11	cg12565681	RHBDF2	0.834	0.053	0.781	1.44E−27	1.68E−27	0.822	0.295	0.527	5.87E−18	5.87E−18
12	cg20564913	FGFRL1	0.837	0.108	0.729	8.94E−28	1.67E−27	0.832	0.222	0.610	1.23E−20	2.87E−20
13	cg25499397	GPR62	0.822	0.076	0.746	8.35E−28	1.67E−27	0.798	0.272	0.527	1.12E−19	1.38E−19
14	cg25813864	RAPGEFL1	0.851	0.064	0.787	2.64E−27	2.64E−27	0.855	0.160	0.695	9.37E−21	2.87E−20

*NA* not associated with any gene

**Table 2:** For the methylation-based signatures: overall diagnostic accuracy, sensitivity, and specificity

1. Low-grade glioma IDH WT versus mutant: for 14 CpG methylation signatures									
Cohort	Dataset	Overall accuracy (%)^a^	Sensitivity (%)^b^		Specificity (%)^c^		Overall error (%)	IDH mutant error (%)	IDH WT error (%)
			IDH mutant	IDH WT	IDH mutant	IDH WT			
TCGA	Training set	100 (266/266)	100 (217/217)	100 (49/49)	100 (49/49)	100 (217/217)	0	0	0
TCGA	Test set	99.62 (263/264)	99.53 (215/216)	100 (48/48)	100 (48/48)	99.53 (215/216)	0.38	0.47	0
TCGA	Combined set	99.81 (529/530)	99.76 (432/433)	100 (97/97)	100 (97/97)	99.76 (432/433)	0.19	0.24	0
GSE58218	Validation dataset	98.5 (192/195)	99.36 (156/157)	94.7 (36/38)	94.7 (36/38)	99.36 (156/157)	1.5	0.64	5.3
GSE48462	Validation dataset	85.8 (48/56)	96.55 (28/29)	74.07 (20/27)	74.07 (20/27)	96.55 (28/29)	14.2	3.4	25.9
2. Diffuse astrocytoma (IDH mutant and non-codeletion of 1p/19q; DA) versus oligodendroglioma (IDH mutant and 1p/19q codeletion; ODG): for 14 CpG methylation signatures									
Cohort	Dataset	Overall accuracy (%)^a^	Sensitivity (%)^b^		Specificity (%)^c^		Overall error (%)	DA error (%)	ODG error (%)
			DA	ODG	DA	ODG			
TCGA	Training set	99.07 (215/217)	98.47 (129/131)	100 (86/86)	100 (86/86)	98.47 (129/131)	0.93	1.53	0
TCGA	Test set	96.29 (208/216)	94.61 (123/130)	98.83 (85/86)	98.83 (85/86)	94.61 (123/130)	3.71	5.39	1.17
TCGA	Combined set	97.69 (423/433)	96.55 (252/261)	99.41 (171/172)	99.41 (171/172)	96.55 (252/261)	2.31	3.45	0.59
GSE58218	Validation dataset	97.5 (153/157)	96.25 (77/80)	98.70 (77/78)	98.70 (77/78)	96.25 (77/80)	2.5	3.75	1.29
GSE48462	Validation dataset	78.57 (22/28)	71.42 (10/14)	85.71 (12/14)	85.71 (12/14)	71.42 (10/14)	21.43	28.58	14.29
3. For GBM IDH WT versus mutant: for 13 CpG methylation signatures									
Cohort	Dataset	Overall accuracy (%)^a^	Sensitivity (%)^b^		Specificity (%)^c^		Overall error (%)	GBM IDH mutant error (%)	GBM IDH WT error (%)
			GBM IDH mutant	GBM IDH WT	GBM IDH mutant	GBM IDH WT			
TCGA	Training set	100 (63/63)	100 (4/4)	100 (59/59)	100 (59/59)	100 (4/4)	0	0	0
TCGA	Test set	98.36 (60/61)	100 (3/3)	98.27 (57/58)	98.27 (57/58)	100 (3/3)	1.64	0	1.73
TCGA	Combined set	99.19 (123/124)	100 (7/7)	99.14 (116/117)	99.14 (116/117)	100 (7/7)	0.81	0	0.86
GSE36278	Validation dataset	96.10 (74/77)	87.5 (14/16)	98.36 (60/61)	98.36 (60/61)	87.5 (14/16)	3.9	12.5	1.64

1. low-grade glioma IDH WT versus mutant, 2. diffuse astrocytoma (DA) versus oligodendroglioma (ODG), 3. GBM IDH WT versus mutant

^a^(the number of samples predicted correctly)/(total number of samples analyzed)×100

^b^(the number of positive samples predicted)/(the number of true positives)×100

^c^(the number of negative samples predicted)/(the number of true negatives)×100

Next, we validated the strength of 14 CpG methylation signatures using the test set (Additional file 1: Table S1). The 14 discriminatory probes were observed to be differentially methylated between LGG IDH mutant and LGG IDH WT in the test set also (Additional file 2: Figure S3A and Additional file 1: Table S3A). The PCA demonstrated that the probes were able to distinguish IDH mutant from the WT group as two distinct clusters (Additional file 2: Figure S3B). Prediction accuracy estimation by 10-fold cross-validation using PAM showed that the 14 CpG probe methylation signatures predicted all IDH mutant LGG samples accurately except one with an error rate of 0.004 (Additional file 2: Figure S3C). Among IDH WT LGG samples, all of them were accurately predicted by the signature (Additional file 2: Figure S3C). Thus, the 14 CpG methylation signatures were able to discriminate between IDH mutant and WT LGG samples with an overall diagnostic accuracy of 99.62% in the test set. The sensitivity of the signature for IDH mutant LGG is 99.53% while for IDH WT LGG is 100%, and the specificity for IDH mutant is 100% whereas for those of the IDH WT, it is 99.53% (Table 2). The 14 CpG methylation signatures, as identified in the training set and validated in the test set, were also used to classify the entire set of TCGA LGG. We found that the 14 discriminatory probes distinguished two groups (Additional file 2: Figure S4A, B, and C) with an overall accuracy of 99.81% (Table 2).

**Fig. 4:**
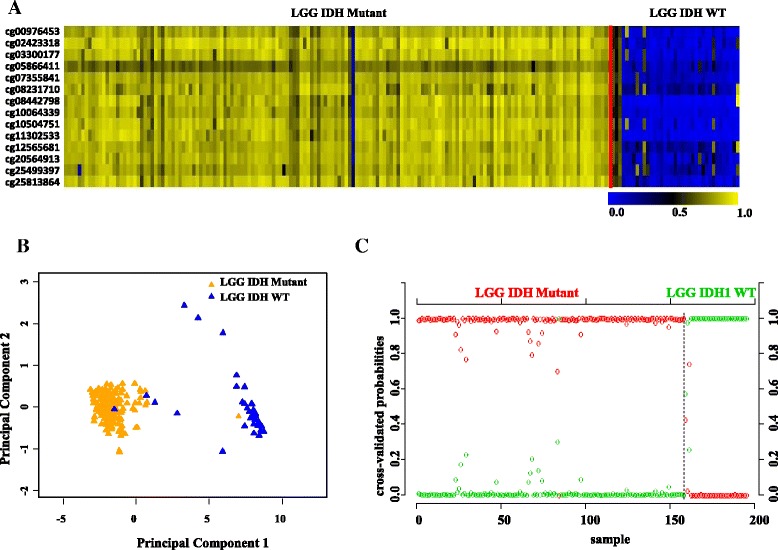
Validation of the 14 CpG methylation signatures of LGG IDH mutant versus WT in an independent validation dataset GSE58218. **a** Heat map of the 14 CpG discriminatory probes identified in PAM analysis in IDH mutant (*n* = 157) and WT (*n* = 38) LGG patient samples in the entire TCGA dataset. A *dual color code* was used where *yellow* indicates more methylation (hypermethylation) and *blue* indicates less methylation (hypomethylation). **b** PCA was performed using *β* (methylation) values of 14 PAM-identified CpG probes between IDH mutant (*n* = 157) and WT (*n* = 38) LGG patient samples in the entire TCGA dataset. A scatter plot is generated using the first two principal components for each sample. The *color code* of the samples is as indicated. **c** The detailed probabilities of 10-fold cross-validation for the samples of training set based on the *β* values of 14 CpG probes are shown. For each sample, its probability as IDH mutant (*red color*) and WT (*green color*) of LGG patient samples is shown and it was predicted by the PAM program as either LGG IDH mutant or WT based on which grade’s probability is higher. The original histological grade of the samples is shown on the *top*

Next, we have also carried out additional validation of 14 CpG methylation signatures using two independent external LGG cohorts (GSE58218 [22] and GSE48462 [23]). In GSE58218, the 14 CpG methylation signatures were able to discriminate IDH mutant from WT LGG samples with an overall diagnostic accuracy of 98.5% (Tables 1 and 2; Fig. 4a–c). Similarly, the 14 CpG methylation signatures were able to discriminate IDH mutant from WT LGG samples with an overall diagnostic accuracy of 85.8% in GSE48462 (Table 2; Additional file 1: Table S3A; Additional file 2: Figure S5A, B, and C). Thus, from these experiments, we conclude that the 14 CpG methylation signatures developed as above distinguished LGG IDH mutant from WT samples with high accuracy.

### 14 CpG probe methylation signatures to classify oligodendrogliomas (ODG) and diffuse astrocytoma (DA): identification and validation

**Fig. 5:**
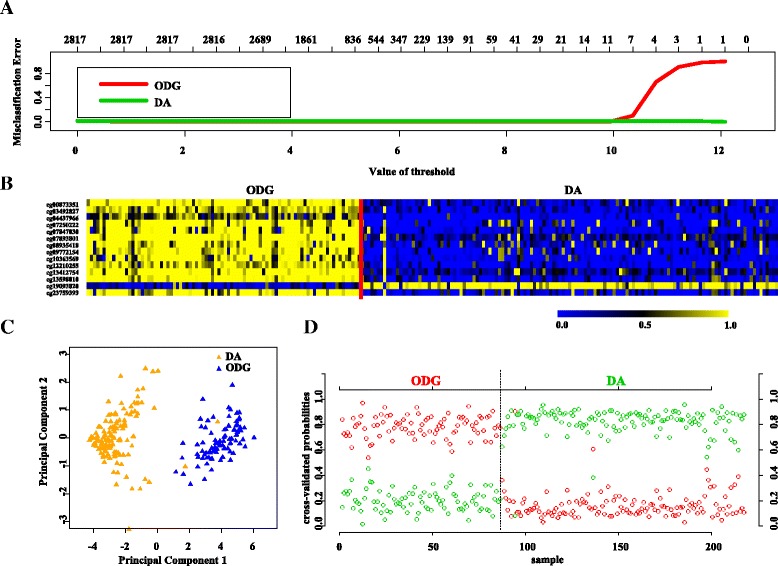
Identification of 14 CpG probe methylation signatures in training set (TCGA) for diffuse astrocytoma (DA) and oligodendroglioma (ODG). **a** Plot demonstrating classification error for 2817 CpG probes from PAM analysis in training set. The threshold value of 9.491 corresponded to 14 discriminatory CpG probes which classified DA (LGG IDH Mut with intact 1p/19q; *n* = 131) and ODG (LGG IDH Mut with 1p/19q codel; *n* = 86) LGG samples with classification error of 0.93%. **b** Heat map of the 14 CpG discriminatory probes identified from the PAM analysis between DA and ODG patient samples in the training set (TCGA). A *dual color code* was used where *yellow* indicates more methylation (hypermethylation) and *blue* indicates less methylation (hypomethylation). **c** PCA was performed using beta (methylation) values of 14 PAM-identified CpG probes between DA (*n* = 131) and WT (*n* = 86) LGG samples in training set. A scatter plot is generated using the first two principal components for each sample. The *color code* of the samples is as indicated. **d** The detailed cross-validation probabilities of 10-fold cross-validation for the samples of training set based on the beta values of 14 CpG probes are shown. For each sample, its probability as ODG (*red color*) and DA (*green color*) is shown and it was predicted by the PAM program as either ODG or DA in LGG samples based on which grade’s probability is higher. The original histological grade of the samples is shown on the *top*

PAM analysis of differentially methylated CpGs (Additional file 1: Table S4) on the training (TCGA) set (Additional file 1: Table S1) identified a set of 14 CpGs to distinguish IDH mutant with 1p/19q codeletion (designated as oligodendroglioma) from LGG IDH mutant with intact 1p/19q loci (designated as diffuse astrocytoma) at a threshold value of 9.491 with minimal error (Fig. 5a, Additional file 2: Figure S1B). The robustness of this probe set was tested by internal cross-validation using SVM, which gave a classification accuracy of 97.67 to 100% and subset validation with an accuracy of 99 to 100% (Additional file 2: Figure S2C and D, respectively; see the Methods section for more detail). The CpG probes that correspond to this signature were found to be hypermethylated in oligodendroglioma compared to diffuse astrocytoma (Fig. 5b and Table 3). Further, upon subjecting the 14 CpG probes to PCA, the two principal components were able to separate these two groups into two distinct clusters (Fig. 5c). Prediction accuracy estimation by 10-fold cross-validation using PAM showed that the 14 CpG probe methylation signatures predicted all oligodendroglioma samples accurately with no error (Fig. 5d). With respect to diffuse astrocytoma, all samples except two were accurately predicted to be diffuse astrocytoma based on the 14 CpG probe methylation signatures with an error rate of 0.0153 (Fig. 5d). Thus, the 14 CpG DNA methylation signatures were able to discriminate oligodendroglioma from diffuse astrocytoma with an overall diagnostic accuracy of 99.07%. The sensitivity of the signature for oligodendroglioma is 100% while for diffuse astrocytoma is 98.47%, and the specificity for oligodendroglioma is 98.47% whereas for those of the diffuse astrocytomas is 100% (Table 2).

**Table 3:** List of the 14 CpG methylation signatures for oligodendroglioma (ODG) versus diffuse astrocytoma (DA) in the training set and validation set (GSE58218)

			Training set (TCGA cohort)					Validation set (GSE58218 cohort)				
No.	CpG ID	Gene name	Average *β* in ODG	Average *β* in DA	∆*β* = (avg *β* in ODG−avg *β* in DA)	*p* value	FDR	Average *β* in ODG	Average *β* in DA	∆*β* = (avg *β* in ODG−avg *β* in DA)	*p* value	FDR
1	cg00873351	CD300LB	0.755	0.227	0.528	1.67E−32	3.9E−32	0.576	0.225	0.351	1.92E−22	2.99E−22
2	cg03492827	NA	0.647	0.192	0.455	8.18E−34	5.73E−33	0.701	0.286	0.415	3.01E−26	2.11E−25
3	cg04437966	FLJ37543	0.500	0.088	0.412	3.99E−33	1.39E−32	0.459	0.102	0.357	2.33E−25	8.16E−25
4	cg07250222	FGFR2	0.753	0.186	0.567	8.68E−30	8.68E−30	0.795	0.301	0.494	5.03E−21	7.04E−21
5	cg07847030	TCF7L1	0.730	0.163	0.567	6.78E−31	1.06E−30	0.798	0.368	0.429	2.62E−19	2.62E−19
6	cg07893801	PLCG1	0.807	0.312	0.495	1.28E−32	3.59E−32	0.737	0.378	0.359	1.61E−20	2.04E−20
7	cg08935418	PTPRN2	0.735	0.231	0.505	6.25E−30	6.73E−30	0.771	0.313	0.458	4.01E−23	7.53E−23
8	cg09772154	FGFR2	0.733	0.194	0.540	5.94E−30	6.73E−30	0.779	0.308	0.472	6.35E−20	6.84E−20
9	cg10363569	PRKAG2	0.675	0.162	0.513	5.1E−30	6.5E−30	0.574	0.196	0.377	5.22E−20	6.09E−20
10	cg12210255	NA	0.662	0.139	0.523	2.53E−33	1.18E−32	0.699	0.237	0.462	1.8E−24	5.05E−24
11	cg13412754	MAPKAP1	0.782	0.311	0.471	4.67E−32	9.34E−32	0.736	0.340	0.396	1.74E−25	8.1E−25
12	cg13598010	NA	0.778	0.175	0.603	1.89E−34	2.65E−33	0.790	0.254	0.536	5.09E−27	7.13E−26
13	cg19093820	GPR156	0.210	0.716	−0.506	3.56E−31	6.23E−31	0.211	0.655	−0.444	4.3E−23	7.53E−23
14	cg23759393	PTPRN2	0.722	0.203	0.518	1.08E−30	1.51E−30	0.790	0.300	0.490	8.76E−24	2.04E−23

*NA* not associated with any gene

Next, we validated the strength of 14 CpG methylation signatures using the test (TCGA) set (Additional file 1: Table S1). The 14 discriminatory probes were observed to be differentially methylated between oligodendrogliomas and diffused astrocytoma similar to as seen in the training set (Additional file 2: Figure S6A and Additional file 1: Table S3B). The PCA demonstrated that the probes were able to distinguish oligodendrogliomas from diffused astrocytoma as two distinct clusters (Additional file 2: Figure S6B). Prediction accuracy estimation by 10-fold cross-validation using PAM showed that the 14 CpG probe methylation signatures predicted all oligodendroglioma samples except one accurately with an error rate of 0.0117 (Additional file 2: Figure S6C). Among diffused astrocytoma, except seven, all samples were accurately predicted by the signature with an error rate of 0.0539 (Additional file 2: Figure S6C). Thus, the 14 CpG methylation signatures were able to discriminate between oligodendroglioma and diffused astrocytoma samples with an overall diagnostic accuracy of 96.29% in the test set. The sensitivity of the signature for oligodendrogliomas is 98.83% while for diffused astrocytoma, it is 94.61%, and the specificity for oligodendrogliomas is 94.61% whereas for diffused astrocytoma, it is 98.83% (Table 2). The 14 CpG methylation signatures, as identified in the training set and validated in the test set, were also used to classify the entire TCGA LGG IDH mutant samples into oligodendroglioma and diffuse astrocytoma samples. We found that the 14 discriminatory probes behaved similar in the classification (Additional file 2: Figure S7A, B and C) with an overall accuracy of 97.69% (Table 2).

**Fig. 6:**
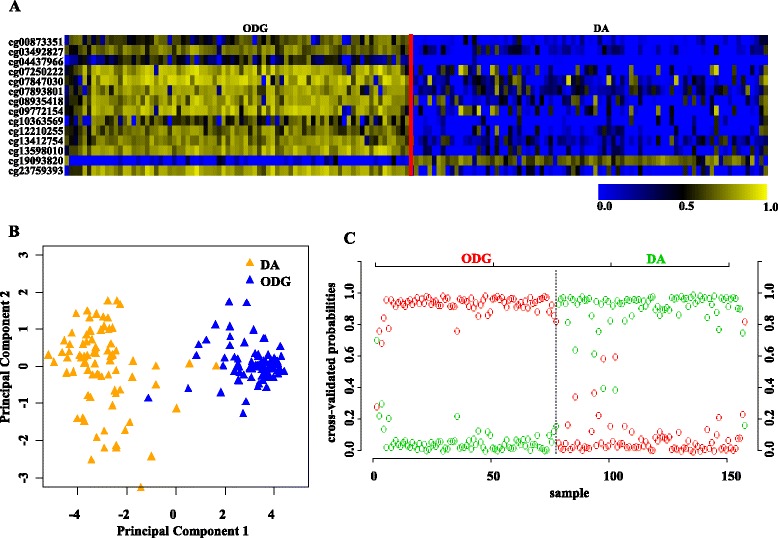
Validation of the 14 CpG methylation signatures of oligodendroglioma (ODG) versus diffuse astrocytoma (DA) in an independent validation dataset GSE58218. **a** Heat map of the 14 CpG discriminatory probes identified in PAM analysis in ODG (*n* = 77) and DA (*n* = 80) LGG patient samples in the entire TCGA dataset. A *dual color code* was used where *yellow* indicates more methylation (hypermethylation) and *blue* indicates less methylation (hypomethylation). **b** PCA was performed using *β* (methylation) values of 14 PAM-identified CpG probes between ODG (*n* = 77) and DA (*n* = 80) LGG patient samples in the entire TCGA dataset. A scatter plot is generated using the first two principal components for each sample. The *color code* of the samples is as indicated. **c** The detailed probabilities of 10-fold cross-validation for the samples of training set based on the *β* values of 14 CpG probes are shown. For each sample, its probability as ODG (*red color*) and DA (*green color*) of LGG patient samples is shown and it was predicted by the PAM program as either LGG DA or ODG based on which grade’s probability is higher. The original histological grade of the samples is shown on the *top*

In addition, we have also carried out additional validation of 14 CpG methylation signatures to distinguish oligodenroglioma from diffuse astrocytoma using two independent external LGG cohorts (GSE58218 and GSE48462). In GSE58218, the 14 CpG methylation signatures were able to discriminate oligodenroglioma from diffuse astrocytoma samples with an overall diagnostic accuracy of 97.5% (Tables 2 and 3; Fig. 6a–c). Similarly, the 14 CpG methylation signatures were also able to discriminate oligodenroglioma from diffuse astrocytoma samples with an overall diagnostic accuracy of 78.57% in GSE48462 (Table 2; Additional file 1: Table S3B; Additional file 2: Figure S8A, B and C). Thus, from these experiments, we conclude that the 14 CpG methylation signatures developed as above distinguished oligodenroglioma from diffuse astrocytoma samples with high accuracy.

### 13 CpG probe methylation signatures to classify IDH mutant from wild type (WT) in glioblastoma (GBM): identification and validation

**Fig. 7:**
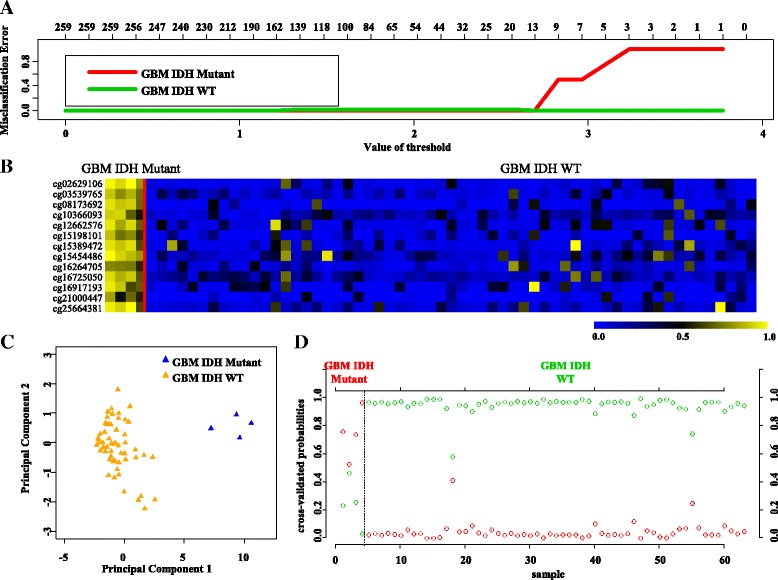
Identification of 13 CpG probe methylation signatures in training set (TCGA) for IDH Mut and WT in GBM. **a** Plot demonstrating classification error for 259 CpG probes from PAM analysis in training set. The threshold value of 2.694 corresponded to 13 discriminatory CpG probes which classified IDH Mut (*n* = 4) and WT (*n* = 59) GBM samples with classification error of 0%. **b** Heat map of the 13 CpG discriminatory probes identified from the PAM analysis between IDH Mut and WT GBM patient samples in the training set (TCGA). A *dual color code* was used where *yellow* indicates more methylation (hypermethylation) and *blue* indicates less methylation (hypomethylation). **c** PCA was performed using beta (methylation) values of 13 PAM-identified CpG probes between IDH Mut (*n* = 4) and WT (*n* = 59) GBM samples in training set. A scatter plot is generated using the first two principal components for each sample. The *color code* of the samples is as indicated. **d** The detailed cross-validation probabilities of 10-fold cross-validation for the samples of training set based on the beta values of 14 CpG probes are shown. For each sample, its probability as IDH Mut (*red color*) and WT (*green color*) GBM samples is shown and it was predicted by the PAM program as either IDH Mut or WT in GBM samples based on which grade’s probability is higher. The original histological grade of the samples is shown on the *top*

PAM analysis of differentially methylated CpGs (Additional file 1: Table S5) in the training (TCGA) set (Additional file 1: Table S1) identified a set of 13 CpGs to distinguish GBM IDH mutant from IDH WT samples at a threshold value of 2.694 with no error (Fig. 7a, Additional file 2: Figure S1C). The robustness of this probe set was tested by internal cross-validation using SVM, which gave a classification accuracy of 100% and subset validation with an accuracy of 100% (Additional file 2: Figure S2E and F, respectively; see the Methods section for more details). The CpG probes of the signature were found to be hypermethylated in IDH mutant GBMs compared to IDH WT GBMs (Fig. 7b and Table 4). Further, upon subjecting the 13 CpG probes to PCA, the two principal components were able to form two distinct clusters for IDH mutant and IDH WT GBMs (Fig. 7c). Prediction accuracy estimation by 10-fold cross-validation using PAM showed that the 13 CpG probe methylation signatures predicted all the samples accurately with no error (Fig. 7d). Similarly, among GBM IDH wild-type samples, all were rightly predicted by the 13 CpG methylation signatures (Fig. 7d). Thus, the 13 CpG DNA methylation signatures were able to discriminate GBM IDH mutant from GBM IDH WT with an overall classification accuracy of 100%. The sensitivity and specificity of the signature for IDH mutant and WT in GBM are 100% (Table 2).

**Table 4:** List of the 13 CpG methylation signatures for GBM IDH mutant versus IDH WT in the training set and validation set (GSE36278)

			Training set (TCGA cohort)					Validation set (GSE36278 cohort)				
No.	CpG ID	Gene name	Average *β* in mutant	Average *β* in WT	∆*β* = (avg *β* in mutant−avg *β* in WT)	*p* value	FDR	Average *β* in mutant	Average *β* in WT	∆*β* = (avg *β* in mutant−avg *β* in WT)	*p* value	FDR
1	cg02629106	PCDP1	0.845	0.113	0.732	0.00093	0.00163	0.76	0.18	0.58	4E−08	2.6E−07
2	cg03539765	LOC144571	0.746	0.127	0.618	0.00093	0.00163	0.63	0.18	0.45	2.7E−07	5.9E−07
3	cg08173692	PRR18	0.791	0.091	0.699	0.00093	0.00163	0.79	0.24	0.54	9.1E−06	1.1E−05
4	cg10366093	YPEL4	0.710	0.189	0.520	0.00102	0.00163	0.67	0.25	0.42	1.2E−06	2.3E−06
5	cg12662576	NA	0.780	0.172	0.608	0.00125	0.00163	0.76	0.29	0.48	2.7E−07	5.9E−07
6	cg15198101	SRRM3	0.695	0.109	0.586	0.00102	0.00163	0.79	0.15	0.64	1.7E−08	2.2E−07
7	cg15389472	GLUL	0.786	0.162	0.624	0.00184	0.00184	0.66	0.24	0.42	8.6E−06	1.1E−05
8	cg15454486	OBFC2A	0.823	0.231	0.592	0.00125	0.00163	0.71	0.32	0.39	3.1E−06	4.5E−06
9	cg16264705	ATP5G2	0.665	0.132	0.533	0.00167	0.00181	0.74	0.23	0.51	2.4E−07	5.9E−07
10	cg16725050	TUBA4B	0.769	0.214	0.556	0.00125	0.00163	0.78	0.32	0.46	2.7E−07	5.9E−07
11	cg16917193	NA	0.736	0.150	0.586	0.00138	0.00163	0.72	0.23	0.49	1.1E−05	1.2E−05
12	cg21000447	CHADL	0.626	0.077	0.549	0.00125	0.00163	0.70	0.19	0.51	3.1E−06	4.5E−06
13	cg25664381	NA	0.808	0.166	0.642	0.00138	0.00163	0.66	0.22	0.44	0.00016	0.00016

*NA* not associated with any gene

**Fig. 8:**
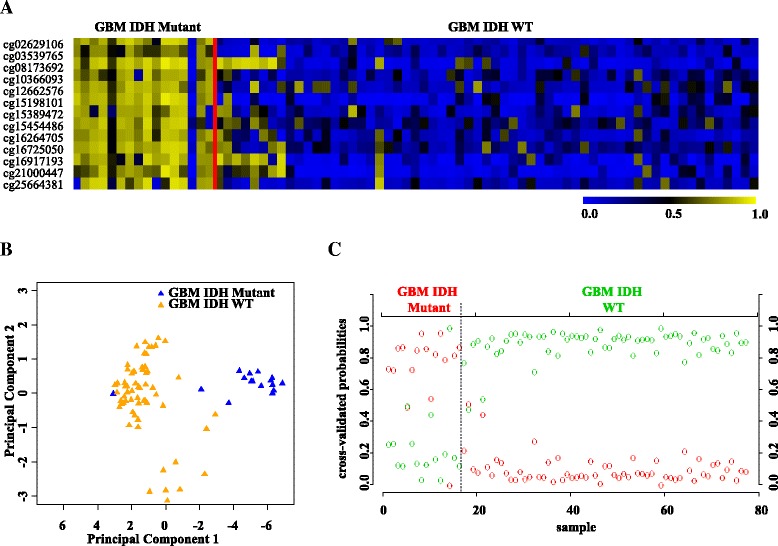
Validation of the 13 CpG methylation signatures of GBM IDH mutant versus WT in an independent validation dataset GSE36278. **a** Heat map of the 13 CpG discriminatory probes identified in PAM analysis in IDH mutant (*n* = 16) and WT (*n* = 61) GBM patient samples in the entire TCGA dataset. A *dual color code* was used where *yellow* indicates more methylation (hypermethylation) and *blue* indicates less methylation (hypomethylation). **b** PCA was performed using *β* (methylation) values of 13 PAM-identified CpG probes between IDH mutant (*n* = 16) and WT (*n* = 61) GBM patient samples in the entire TCGA dataset. A scatter plot is generated using the first two principal components for each sample. The *color code* of the samples is as indicated. **c** The detailed probabilities of 10-fold cross-validation for the samples of training set based on the *β* values of 13 CpG probes are shown. For each sample, its probability as IDH mutant (*red color*) and WT (*green color*) of GBM patient samples is shown and it was predicted by the PAM program as either GBM IDH mutant or WT based on which grade’s probability is higher. The original histological grade of the samples is shown on the *top*

Next, we validated the strength of 13 CpG methylation signatures using the test set (Additional file 1: Table S1). The 13 discriminatory probes were observed to be differentially methylated between GBM IDH mutant and GBM IDH WT in the test set also (Additional file 2: Figure S9A and Additional file 1: Table S3C). The PCA demonstrated that the probes were able to distinguish IDH mutant from the WT group as two distinct clusters (Additional file 2: Figure S9B). Prediction accuracy estimation by 10-fold cross-validation using PAM showed that the 13 CpG methylation signatures predicted all IDH mutant GBM samples accurately with no error rate (Additional file 2: Figure S9C). Among IDH WT GBM samples, all samples except one were accurately predicted by the signature with an error rate of 0.0173 (Additional file 2: Figure S9C). Thus, the 13 CpG methylation signatures were able to discriminate IDH mutant from WT GBM samples with an overall diagnostic accuracy of 98.36% in the test set. The sensitivity of the signature for IDH mutant GBM is 100% while for IDH WT GBM is 98.27%, and the specificity for IDH mutant is 98.27% whereas for those of the IDH WT, it is 100% (Table 2). The 13 CpG methylation signatures, as identified in the training set and validated in the test set, were also used to classify the entire set of TCGA GBM set (117 IDH WT samples and 7 IDH mutant samples). We found that the 13 discriminatory probes distinguished two groups (Additional file 2: Figure S10A, B, and C) with an overall accuracy of 99.19% (Table 2). Further, we have also carried out additional validation of 13 CpG methylation signatures to distinguish GBM IDH mutant from WT samples using an independent external GBM cohort (GSE36278 [24]). Analysis revealed that the 13 CpG methylation signatures were able to discriminate GBM IDH mutant from WT samples with an overall diagnostic accuracy of 96.10% (Tables 2 and 4; Fig. 8a–c). Thus, from these experiments, we conclude that the 13 CpG methylation signatures developed as above distinguished GBM IDH mutant from WT samples with high accuracy.

### Molecular analysis of discordant samples

While the DNA methylation signatures were able to distinguish different diffuse glioma subtypes, it also identified a fraction of samples as discordant. It is of our interest to find out the accurate molecular nature of these samples in order to assess the true nature of them. While we could use TCGA cohort for this purpose as it had all relevant histological and molecular markers, external validation cohorts could not be subjected to molecular discordant analysis as they do not have these features. In the classification of LGG IDH mutant from IDH WT, the 14 CpG signatures identified one IDH mutant LGG sample in the test set as discordant. We carried out a careful assessment of the molecular markers of this sample using c-Bioportal (http://www.cbioportal.org/) from the TCGA dataset. For this purpose, we analyzed TP53 mutation, ATRX loss, and 1p/19q codeletion status of all the samples (Additional file 1: Table S6, Table S7 A, B, and C, and Table S8). As per 2016 WHO CNS tumor classification, all LGG IDH mutant samples that have 1p/19q codeletion are designated as oligodendroglioma and those with intact 1p/19q loci and enriched for TP53 mutation/ATRX loss are designated as diffuse astrocytoma. The LGG IDH mutant discordant sample had intact 1p/19q, WT TP53, and ATRX genes indicating that this sample is not an oligodendroglioma. The presence of WT TP53 and ATRX genes raises the possibility of it not being a diffuse astrocytoma. Interestingly, additional analysis revealed that the discordant sample is indeed carrying WT IDH as per DNA sequencing even though IDH antibody-based scoring classified it as IDH mutant. Therefore, it appears that IDH mutation scoring by IHC could be an error as evidenced by DNA sequencing and that the 14 CpG methylation signatures are able classify the LGGs more accurately.

In the classification of LGG oligodendroglioma from LGG diffuse astrocytoma, 14 CpG probe methylation signatures identified ten samples as discordant which did not match the WHO 2016 tumor grading. In order to understand the true status of the discordant samples, we analyzed the clinical information and molecular markers using c-Bioportal (http://www.cbioportal.org/) from the TCGA dataset. For this purpose, we analyzed TP53 mutation, ATRX mutation, and 1p/19q codeletion status in DA, ODG, and discordant samples of LGG (Additional file 1: Table S6, Table S7 A, B, and C, and Table S8). Based on the WHO 2016 CNS tumor classification, IDH mutant LGGs having intact 1p/19q with an enrichment of TP53 mutation and ATRX loss are classified as diffuse astrocytoma. IDH mutant LGG samples with 1p/19q codeletion are classified as oligodendroglioma. The analysis of discordant samples for the molecular markers and histological features revealed some interesting findings. While the single ODG discordant sample had 1p/19q codeletion and WT TP53/ATRX genes, this sample was identified as oligoastrocytoma as per histology. Among nine DA discordant samples, while all of them had intact 1p/19q loci, a majority of them were found to have WT TP53/ATRX genes.

In the classification of GBM IDH mutant from IDH WT, the 13 CpG probe methylation signatures identified one GBM IDH WT sample as discordant. In order to understand the true nature of the discordant sample, we analyzed the clinical information and molecular markers using c-Bioportal (http://www.cbioportal.org/) from the TCGA dataset (Additional file 1: Table S6, Table S8, and Table S9 A and B). The discordant GBM IDH WT sample had WT IDH gene as per both immunohistochemical staining and DNA sequencing. However, this sample had no amplification of EGFR locus with an intact PTEN gene, unlike what is expected for a IDH WT GBM sample.

## Discussion

Glioma is the most common and highly malignant primary brain tumor. The 2007 WHO classification of the glioma tumors was majorly based on microscopic appearance of cell type and histopathological markers largely segregating into three subtypes such as astrocytoma, oligodendroglioma, and oligoastrocytoma (mixed) [3]. With the advent of the high-throughput technologies, comprehensive understanding of the heterogeneous genetic and epigenetic landscape of both glioblastoma and the low grades became vibrant [25, 26]. The histopathological grading of glioma tumors could be subjected to inter-observer variation which would lead to misclassification with a potential possibility of not providing the right kind of treatment [27]. To combat this shortcoming, several groups including work from our laboratory carried out extensive studies and have identified several prognostic markers and molecular signatures based on mRNA, miRNA, and DNA methylation that would aid in better classification and identifying best choice of therapy [10–13, 15, 28–31].

The meeting by the International Society of Neuropathology held in Haarlem, Netherland, established guidelines for how to incorporate molecular findings into brain tumor diagnosis thereby setting the platform for a major revision of the 2007 CNS WHO classification [32]. The current updated version is summarized in the 2016 CNS WHO classifications [19]. In this study, using TCGA 450K DNA methylation data, we developed methylation signatures that could distinguish different classes of diffuse glioma with high accuracy. The signatures developed in this study using TCGA data are also validated extensively using TCGA data as well as independent datasets.

Infinium HumanMethylation450K BeadChip array data for astrocytoma (grade II, III, and IV/GBM), oligodendroglioma, and oligoastrocytoma tumor samples from TCGA dataset was used in this study. By using PAM, we have successfully developed and validated DNA methylation signatures to distinguish LGG IDH mutant from LGG IDH wild-type samples, LGG IDH mutant samples into diffuse astrocytoma and IDH mutant GBM from the IDH WT GBMs. The signatures classified these groups with very high accuracy and also validated successfully in multiple independent datasets. We also used PCA to test the ability of signatures to divide the two groups in comparison into two distinct classes. Further, the 10-fold cross-validation using PAM identified the discordant samples, which upon further analysis revealed that majority of misclassified samples were indeed due to inadequacies of the current methods used for classification.

Thus, the present study enabled us to identify DNA methylation fingerprint for each of the groups in comparison (LGG IDH1 WT versus mutant, ODG versus DA, and GBM IDH mutant versus WT). The 2016 WHO classification system fails to classify some samples accurately in occasions like absence of certain molecular markers, errors due to antibody-based scoring, and intra-tumoral heterogeneity. We believe that DNA methylation signatures based on whole tumor developed in this study could complement the 2016 WHO classification of diffuse glioma subtypes.

## Conclusions

In conclusion, we were able to classify diffuse glioma subtypes with high accuracy. The discordant samples identified by the methylation signature were found to be either due to technical errors or mixed histological types. More importantly, we believe that the high levels of intra-tumoral heterogeneity reported in glioma could also be a reason for their misclassification [7, 27]. Collectively, our study indicates that the methylation-based molecular profiles in combination with the revised 2016 WHO CNS tumor classification guidelines might be able to classify the samples more precisely.

## Methods

### Tumor samples and clinical details

Glioma TCGA dataset was used for this study. Methylation data for histologically defined WHO classification glioma types, which include astrocytoma (*n* = 197), oligoastrocytoma (*n* = 136), oligodendroglioma (*n* = 197), and glioblastoma (*n* = 124) samples, was used. Samples were then segregated according to the WHO 2016 CNS tumor IHC-based grading classification into three distinct groups, namely 1. lower grade glioma IDH wild-type and mutant (LGG IDH WT and mutant), 2. lower grade glioma IDH mutant with intact 1p/19q termed as diffuse astrocytoma and with 1p/19q codeletion termed as oligodendroglioma (DA and ODG), and 3. glioblastoma IDH mutant and wild type (GBM IDH WT and mutant). The clinical information for the same was also procured from TCGA.

With an aim to identify methylation differences between the diffuse glioma subtypes (based on IDH mutation and 1p/19q codeletion status) of each group, a supervised machine learning approach through PAM (Prediction Analysis of Microarrays) [20] was used. For this purpose, the first step was to identify significantly differentially methylated CpG probes between lower grade glioma IDH WT and mutant, between DA and ODG, and between GBM IDH mutant and WT which are described in details below.

### Identification of differentially methylated CpGs

In this study, three different comparisons were carried out—1. LGG: IDH mutant versus WT, 2. LGG IDH mutant: 1p/19q codel (ODG) versus non-codel (DA), and 3. GBM: IDH mutant versus WT. For the first comparison between LGG IDH mutant and WT, we have performed a Wilcoxon-rank sum test between IDH mutant and WT which yielded 269,442 CpG probes significantly (FDR ≤0.0001) differentially methylated in mutant versus WT. Next, a stringent cutoff of 0.4 absolute Δ*β* value was applied that showed 9,554 significantly differentially methylated (26 CpGs were hypomethylated and 9528 CpGs were hypermethylated in IDH mutant LGG; Additional file 1: Table S2) CpG probes in mutant as compared to WT IDH LGG patients. Firstly, the TCGA 450K human methylation dataset for LGG patients with IDH mutation (*n* = 433) and LGG patients with WT IDH (*n* = 97) was randomized and 50% of each of the two classes formed the training set, and the remaining 50% was used as the test set. We randomized TCGA dataset ten times to obtain ten different training sets and their corresponding test sets. After performing PAM on each of the ten training sets, the training set that gave least error with minimum number of CpGs was selected for further studies. This process gave a set of 14 discriminatory CpG probes which were further tested through SVM and subset analysis before testing on the test set and external validation sets (Fig. 2; Table 1).

Similarly, analysis was carried out for LGG IDH mutant cohort with and without 1p/19q codeletion (ODG and DA, respectively) patients (Fig. 2). For this comparison, between LGG IDH mutant 1p/19q codel (ODG) and non-codel (DA), we have performed a Wilcoxon-rank sum test which yielded 160,288 CpG probes significantly differentially methylated in ODG versus DA. Next, a stringent cutoff of 0.2 absolute Δ*β* value was applied that showed 2817 significantly differentially methylated (627 CpGs were hypomethylated and 2190 CpGs were hypermethylated in ODG; Additional file 1: Table S4) CpG probes in mutant as compared to WT IDH LGG patients. The TCGA 450K human methylation dataset for LGG patients with 1p/19q codel (*n* = 172) and non-codel (*n* = 261) was randomized and 50% of each of the two classes formed the training set, and the remaining 50% was used as the test set. We randomized TCGA dataset ten times to obtain ten different training sets and their corresponding test sets. After performing PAM on each of the ten training sets, the training set that gave least error with minimum number of CpGs was selected for further studies. This process gave a set of 14 discriminatory CpG probes which were further tested through SVM and subset analysis before testing on the test set and external validation set (Fig. 2; Table 3).

Likewise, the same work flow was followed to identify a methylation-based signature that could distinguish the GBM IDH WT from mutant samples (Fig. 2). In this comparison, between GBM IDH mutant and WT patient samples, we have performed a Wilcoxon-rank sum test which yielded 69,669 CpG probes significantly differentially methylated in mutant versus WT. Next, a stringent cutoff of 0.2 absolute Δ*β* value was applied that showed 259 significantly differentially methylated (33 CpGs were hypomethylated and 226 CpGs were hypermethylated in mutant; Additional file 1: Table S5) CpG probes in mutant as compared to WT IDH GBM patients. The TCGA 450K human methylation dataset for GBM patients with IDH mutation (*n* = 7) and WT (*n* = 117) was randomized and 50% of each of the two classes formed the training set, and the remaining 50% was used as the test set. We randomized TCGA dataset ten times to obtain ten different training sets and their corresponding test sets. After performing PAM on each of the ten training sets, the training set that gave least error with minimum number of CpGs was selected for further studies. This process gave a set of 13 discriminatory CpG probes which were further tested through SVM and subset analysis before testing on the test set and external validation set (Fig. 2; Table 4).

### Prediction Analysis of Microarray (PAM)

To identify a list of a minimal set of signatory probes from the significantly differentially methylated CpGs between each compared groups, Prediction Analysis of Microarrays (PAM) using the package pamr available in R software (version 3.1.0) were applied. PAM uses nearest shrunken centroid method for classifying samples. This method “shrinks” each of the class centroids towards the overall centroid by the threshold. In case of selecting a signature, it is ideal to choose a threshold value that would achieve a set of minimum number of genes with maximum accuracy thereby least error. For preparing input files for PAM analysis, the list of significantly methylated probes between each compared groups across all the tumor samples was randomized and 50% of each of the two classes formed the training set, and the remaining 50% was used as the test set. This randomization was performed ten times which resulted into ten different compositions of training set and their corresponding test set. Thereafter, each of these ten training sets was subjected to PAM analysis that uses 10-fold cross-validation to identify a predictive signature. Ten different training sets that were used to construct the PAM classifier resulted in ten non-identical predictive signatures, one for each iteration. The most promising signature which had the maximum training and test set accuracies was chosen. We also performed an internal cross-validation on the training set of the most promising signature as predicted by PAM.

### Internal cross-validation using Support Vector Machine (SVM) and random subset sampling

For internal cross-validation, we have used Support Vector Machine (SVM) [21]. Many prediction methods use SVM for classification of dataset into two or more classes. For a given set of binary classes training examples, SVM can map the input space into higher dimensional space and seek a hyperplane to separate the positive data examples from the negative ones with the largest margin. SVM-based internal cross-validation is used for the training sets of 1. LGG IDH mutant versus WT, 2. diffuse astrocytoma versus oligodendroglioma, and 3. GBM IDH mutant versus WT. For each of the abovementioned cases, the samples were divided randomly into five subgroups containing equal number of the respective samples. These five subgroups of each cases, example LGG IDH mutant and WT, were made into five groups where each group contained one subgroup of LGG IDH mutant and one subgroup of LGG IDH WT samples. Consequently, one group of LGG IDH WT plus LGG IDH mutant was considered as a test set while the rest four groups were considered as training set and this is referred to as a “fold.” In this way, SVM models were built five times to give fivefolds, wherein every group was considered as a test set and the remaining groups as training set. The accuracy for each fold was checked by this method.

The predictive accuracy of the three signatures was also analyzed in a subset of the following cases: 1. LGG IDH mutant (217) versus WT (*n* = 49), 2. diffuse astrocytoma (*n* = 131) versus oligodendroglioma (*n* = 86), and 3. GBM IDH mutant (*n* = 4) versus WT (*n* = 59) by random subset sampling. PAM was used to predict the respective accuracies in the random subset sampling.

### Principal component analysis

Principal component analysis (PCA) uses orthogonal transformation to convert a set of variables into a set of values of linearly uncorrelated variables that are called principal components. The number of principal components can be less than or equal to the number of original variables. The first two principal components account for the largest possible variation in the dataset. PCA was performed using R package (version 3.1.0), on the training and test sets to know how well the identified methylation signature classifies LGG IDH mutant and WT.

This process was repeated for identifying a methylation signature between IDH mutant DA and ODG and between GBM IDH mutant and WT (a cutoff of 0.2 absolute ∆*β* was used here to identify significantly differently methylated probes between the two classes).

## Additional files

Additional file 1: Table S1. Sample size and diffuse glioma subsets of various cohorts used in this study. **Table S2.** List of differentially methylated CpGs between LGG IDH mutant and WT used as PAM input in the training (TCGA) set. **Table S3A.** List of the 14 CpG methylation signatures for LGG IDH mutant versus IDH WT in the test set (TCGA) and validation set (GSE48462). **Table S3B.** List of the 14 CpG methylation signatures for oligodendroglioma (ODG) versus diffuse astrocytoma (DA) in the test set (TCGA) and validation set (GSE48462). **Table S4.** List of differentially methylated CpGs between oligodendroglioma and diffuse astrocytoma used as PAM input in the training (TCGA) set. **Table S5.** List of 259 differentially methylated CpG probes between GBM IDH mutant and WT used as PAM input in the training (TCGA) set. **Table S6.** Molecular analysis of discordant samples identified by CpG methylation signatures. **Table S7A.** Molecular status for IDH, TP53, ATRX, and 1p/19q in LGG samples from TCGA used in this study. **Table S7B.** Molecular status for IDH, TP53, ATRX, and 1p/19q in LGG samples from GSE58218 used in this study. **Table S7C.** Molecular status for IDH, TP53, ATRX, and 1p/19q in LGG samples from GSE48462 used in this study. **Table S8.** Patient IDs of the discordant samples derived from all datasets used in this study. **Table S9A.** Molecular status of IDH, TP53, ATRX, EGFR, and PTEN in GBM samples from TCGA dataset used in this study. **Table S9B.** Molecular status of IDH, TP53, ATRX, EGFR, and PTEN in GBM samples from GSE38278 dataset used in this study. (ZIP 1601 kb)

Additional file 2: Has ten additional figures and their corresponding figure legends. (PPTX 830 kb)

## Abbreviations

AA: Anaplastic astrocytoma
DA: Diffuse astrocytoma
GBM: Glioblastoma
IDH: Isocitrate dehydrogenase
ODG: Oligodendroglioma
PAM: Prediction Analysis of Microarray
PCA: Principal component analysis
SVM: Support Vector Machine
TCGA: The Cancer Genome Atlas
WHO: World Health Organization
