# Supplementary material for: DNA methylation signatures for 2016 WHO classification subtypes of diffuse gliomas
Source: Clin Epigenetics. 2017 Apr 4;9:32. doi: 10.1186/s13148-017-0331-9 (PMC5379538; doi:10.1186/s13148-017-0331-9)
Supplement: Supplementary file 2 — Has ten additional figures and their corresponding figure legends. (PPTX 830 kb) [file 13148_2017_331_MOESM2_ESM.pptx]

## Slide 1
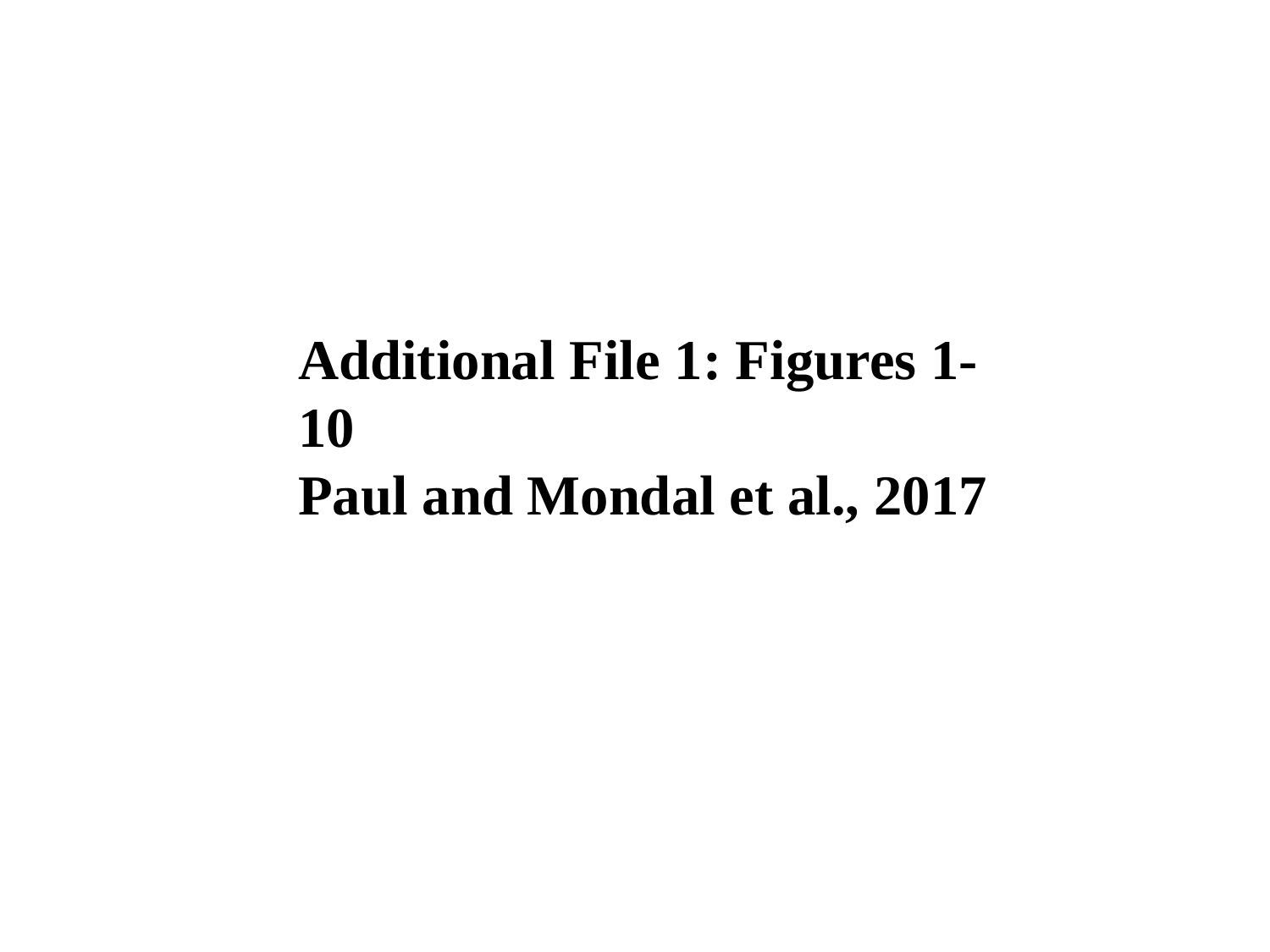

Additional File 1: Figures 1-10
Paul and Mondal et al., 2017

## Slide 2
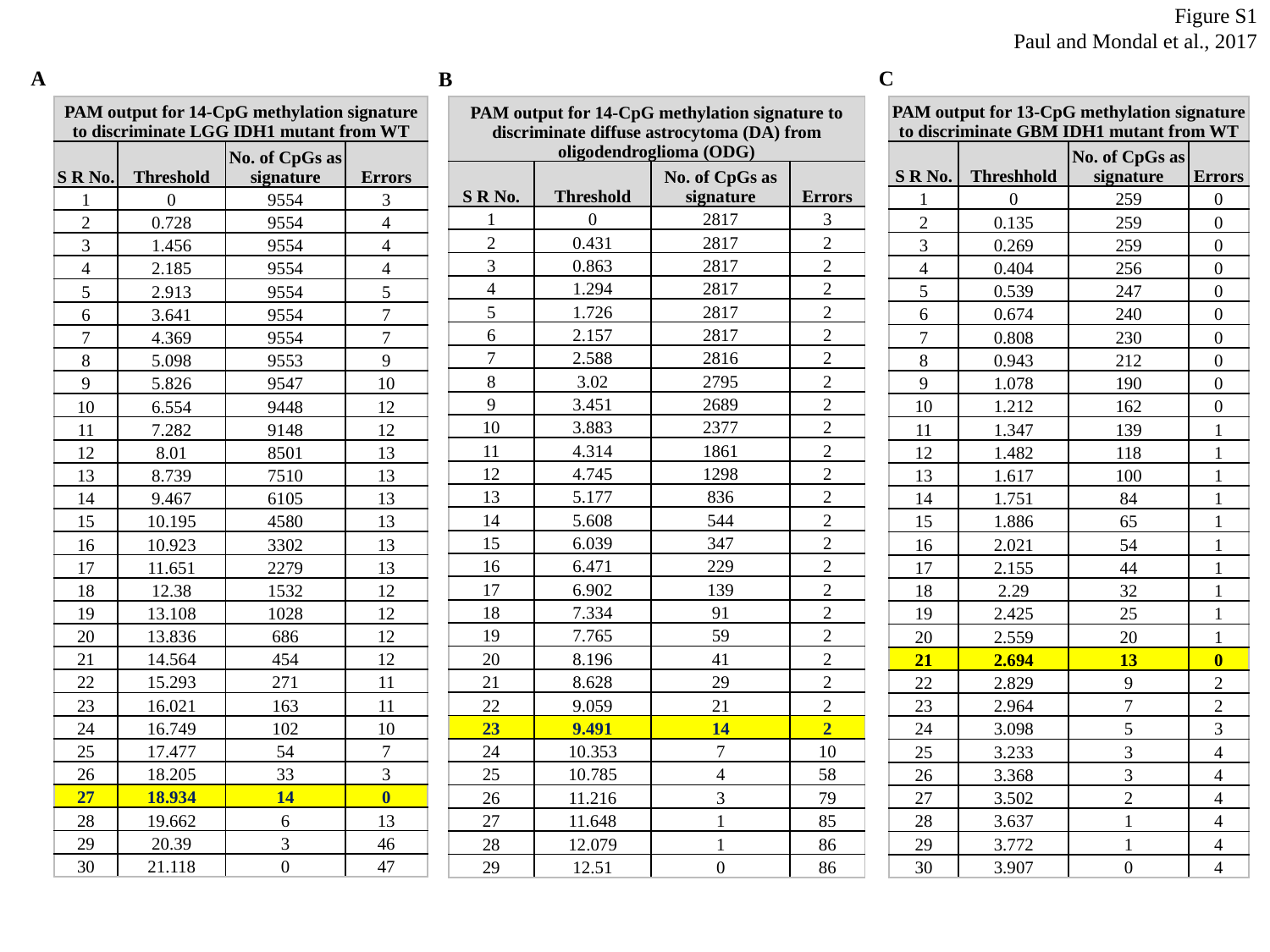

Figure S1
Paul and Mondal et al., 2017
C
A
B
| PAM output for 13-CpG methylation signature to discriminate GBM IDH1 mutant from WT | | | |
| --- | --- | --- | --- |
| S R No. | Threshhold | No. of CpGs as signature | Errors |
| 1 | 0 | 259 | 0 |
| 2 | 0.135 | 259 | 0 |
| 3 | 0.269 | 259 | 0 |
| 4 | 0.404 | 256 | 0 |
| 5 | 0.539 | 247 | 0 |
| 6 | 0.674 | 240 | 0 |
| 7 | 0.808 | 230 | 0 |
| 8 | 0.943 | 212 | 0 |
| 9 | 1.078 | 190 | 0 |
| 10 | 1.212 | 162 | 0 |
| 11 | 1.347 | 139 | 1 |
| 12 | 1.482 | 118 | 1 |
| 13 | 1.617 | 100 | 1 |
| 14 | 1.751 | 84 | 1 |
| 15 | 1.886 | 65 | 1 |
| 16 | 2.021 | 54 | 1 |
| 17 | 2.155 | 44 | 1 |
| 18 | 2.29 | 32 | 1 |
| 19 | 2.425 | 25 | 1 |
| 20 | 2.559 | 20 | 1 |
| 21 | 2.694 | 13 | 0 |
| 22 | 2.829 | 9 | 2 |
| 23 | 2.964 | 7 | 2 |
| 24 | 3.098 | 5 | 3 |
| 25 | 3.233 | 3 | 4 |
| 26 | 3.368 | 3 | 4 |
| 27 | 3.502 | 2 | 4 |
| 28 | 3.637 | 1 | 4 |
| 29 | 3.772 | 1 | 4 |
| 30 | 3.907 | 0 | 4 |
| PAM output for 14-CpG methylation signature to discriminate diffuse astrocytoma (DA) from oligodendroglioma (ODG) | | | |
| --- | --- | --- | --- |
| S R No. | Threshold | No. of CpGs as signature | Errors |
| 1 | 0 | 2817 | 3 |
| 2 | 0.431 | 2817 | 2 |
| 3 | 0.863 | 2817 | 2 |
| 4 | 1.294 | 2817 | 2 |
| 5 | 1.726 | 2817 | 2 |
| 6 | 2.157 | 2817 | 2 |
| 7 | 2.588 | 2816 | 2 |
| 8 | 3.02 | 2795 | 2 |
| 9 | 3.451 | 2689 | 2 |
| 10 | 3.883 | 2377 | 2 |
| 11 | 4.314 | 1861 | 2 |
| 12 | 4.745 | 1298 | 2 |
| 13 | 5.177 | 836 | 2 |
| 14 | 5.608 | 544 | 2 |
| 15 | 6.039 | 347 | 2 |
| 16 | 6.471 | 229 | 2 |
| 17 | 6.902 | 139 | 2 |
| 18 | 7.334 | 91 | 2 |
| 19 | 7.765 | 59 | 2 |
| 20 | 8.196 | 41 | 2 |
| 21 | 8.628 | 29 | 2 |
| 22 | 9.059 | 21 | 2 |
| 23 | 9.491 | 14 | 2 |
| 24 | 10.353 | 7 | 10 |
| 25 | 10.785 | 4 | 58 |
| 26 | 11.216 | 3 | 79 |
| 27 | 11.648 | 1 | 85 |
| 28 | 12.079 | 1 | 86 |
| 29 | 12.51 | 0 | 86 |
| PAM output for 14-CpG methylation signature to discriminate LGG IDH1 mutant from WT | | | |
| --- | --- | --- | --- |
| S R No. | Threshold | No. of CpGs as signature | Errors |
| 1 | 0 | 9554 | 3 |
| 2 | 0.728 | 9554 | 4 |
| 3 | 1.456 | 9554 | 4 |
| 4 | 2.185 | 9554 | 4 |
| 5 | 2.913 | 9554 | 5 |
| 6 | 3.641 | 9554 | 7 |
| 7 | 4.369 | 9554 | 7 |
| 8 | 5.098 | 9553 | 9 |
| 9 | 5.826 | 9547 | 10 |
| 10 | 6.554 | 9448 | 12 |
| 11 | 7.282 | 9148 | 12 |
| 12 | 8.01 | 8501 | 13 |
| 13 | 8.739 | 7510 | 13 |
| 14 | 9.467 | 6105 | 13 |
| 15 | 10.195 | 4580 | 13 |
| 16 | 10.923 | 3302 | 13 |
| 17 | 11.651 | 2279 | 13 |
| 18 | 12.38 | 1532 | 12 |
| 19 | 13.108 | 1028 | 12 |
| 20 | 13.836 | 686 | 12 |
| 21 | 14.564 | 454 | 12 |
| 22 | 15.293 | 271 | 11 |
| 23 | 16.021 | 163 | 11 |
| 24 | 16.749 | 102 | 10 |
| 25 | 17.477 | 54 | 7 |
| 26 | 18.205 | 33 | 3 |
| 27 | 18.934 | 14 | 0 |
| 28 | 19.662 | 6 | 13 |
| 29 | 20.39 | 3 | 46 |
| 30 | 21.118 | 0 | 47 |

## Slide 3
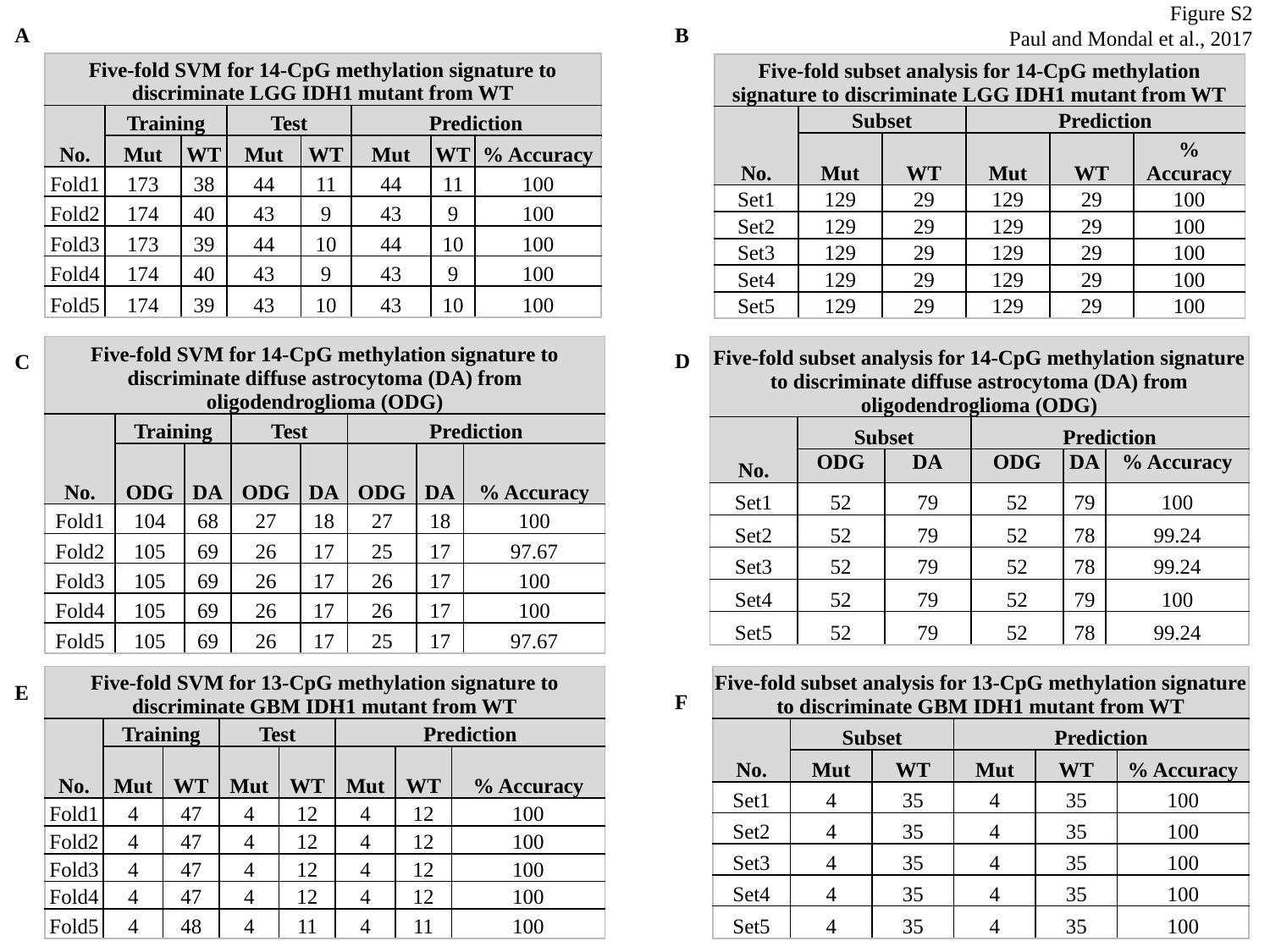

Figure S2
Paul and Mondal et al., 2017
A
B
| Five-fold SVM for 14-CpG methylation signature to discriminate LGG IDH1 mutant from WT | | | | | | | |
| --- | --- | --- | --- | --- | --- | --- | --- |
| No. | Training | | Test | | Prediction | | |
| | Mut | WT | Mut | WT | Mut | WT | % Accuracy |
| Fold1 | 173 | 38 | 44 | 11 | 44 | 11 | 100 |
| Fold2 | 174 | 40 | 43 | 9 | 43 | 9 | 100 |
| Fold3 | 173 | 39 | 44 | 10 | 44 | 10 | 100 |
| Fold4 | 174 | 40 | 43 | 9 | 43 | 9 | 100 |
| Fold5 | 174 | 39 | 43 | 10 | 43 | 10 | 100 |
| Five-fold subset analysis for 14-CpG methylation signature to discriminate LGG IDH1 mutant from WT | | | | | |
| --- | --- | --- | --- | --- | --- |
| No. | Subset | | Prediction | | |
| | Mut | WT | Mut | WT | % Accuracy |
| Set1 | 129 | 29 | 129 | 29 | 100 |
| Set2 | 129 | 29 | 129 | 29 | 100 |
| Set3 | 129 | 29 | 129 | 29 | 100 |
| Set4 | 129 | 29 | 129 | 29 | 100 |
| Set5 | 129 | 29 | 129 | 29 | 100 |
| Five-fold SVM for 14-CpG methylation signature to discriminate diffuse astrocytoma (DA) from oligodendroglioma (ODG) | | | | | | | |
| --- | --- | --- | --- | --- | --- | --- | --- |
| No. | Training | | Test | | Prediction | | |
| | ODG | DA | ODG | DA | ODG | DA | % Accuracy |
| Fold1 | 104 | 68 | 27 | 18 | 27 | 18 | 100 |
| Fold2 | 105 | 69 | 26 | 17 | 25 | 17 | 97.67 |
| Fold3 | 105 | 69 | 26 | 17 | 26 | 17 | 100 |
| Fold4 | 105 | 69 | 26 | 17 | 26 | 17 | 100 |
| Fold5 | 105 | 69 | 26 | 17 | 25 | 17 | 97.67 |
| Five-fold subset analysis for 14-CpG methylation signature to discriminate diffuse astrocytoma (DA) from oligodendroglioma (ODG) | | | | | |
| --- | --- | --- | --- | --- | --- |
| No. | Subset | | Prediction | | |
| | ODG | DA | ODG | DA | % Accuracy |
| Set1 | 52 | 79 | 52 | 79 | 100 |
| Set2 | 52 | 79 | 52 | 78 | 99.24 |
| Set3 | 52 | 79 | 52 | 78 | 99.24 |
| Set4 | 52 | 79 | 52 | 79 | 100 |
| Set5 | 52 | 79 | 52 | 78 | 99.24 |
D
C
| Five-fold SVM for 13-CpG methylation signature to discriminate GBM IDH1 mutant from WT | | | | | | | |
| --- | --- | --- | --- | --- | --- | --- | --- |
| No. | Training | | Test | | Prediction | | |
| | Mut | WT | Mut | WT | Mut | WT | % Accuracy |
| Fold1 | 4 | 47 | 4 | 12 | 4 | 12 | 100 |
| Fold2 | 4 | 47 | 4 | 12 | 4 | 12 | 100 |
| Fold3 | 4 | 47 | 4 | 12 | 4 | 12 | 100 |
| Fold4 | 4 | 47 | 4 | 12 | 4 | 12 | 100 |
| Fold5 | 4 | 48 | 4 | 11 | 4 | 11 | 100 |
| Five-fold subset analysis for 13-CpG methylation signature to discriminate GBM IDH1 mutant from WT | | | | | |
| --- | --- | --- | --- | --- | --- |
| No. | Subset | | Prediction | | |
| | Mut | WT | Mut | WT | % Accuracy |
| Set1 | 4 | 35 | 4 | 35 | 100 |
| Set2 | 4 | 35 | 4 | 35 | 100 |
| Set3 | 4 | 35 | 4 | 35 | 100 |
| Set4 | 4 | 35 | 4 | 35 | 100 |
| Set5 | 4 | 35 | 4 | 35 | 100 |
E
F

## Slide 4
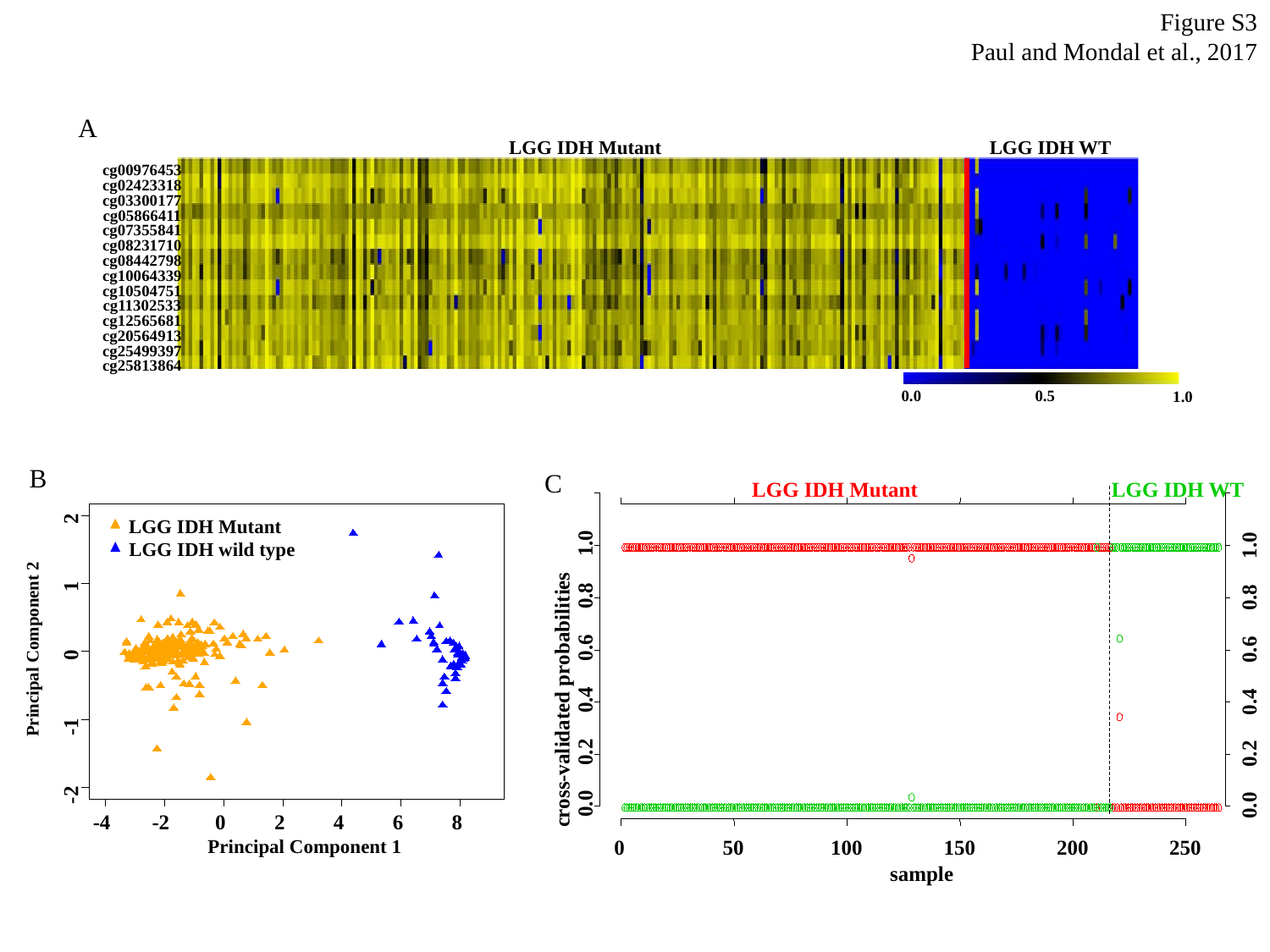

Figure S3
Paul and Mondal et al., 2017
A
LGG IDH Mutant
LGG IDH WT
cg00976453
cg02423318
cg03300177
cg05866411
cg07355841
cg08231710
cg08442798
cg10064339
cg10504751
cg11302533
cg12565681
cg20564913
cg25499397
cg25813864
0.0
0.5
1.0
B
C
LGG IDH Mutant
LGG IDH WT
1.0
1.0
0.8
0.8
0.6
0.6
cross-validated probabilities
0.4
0.4
0.2
0.2
0.0
0.0
0
50
100
150
200
250
sample
2
LGG IDH Mutant
LGG IDH wild type
1
Principal Component 2
0
-1
-2
-4
-2
0
2
4
6
8
Principal Component 1

## Slide 5
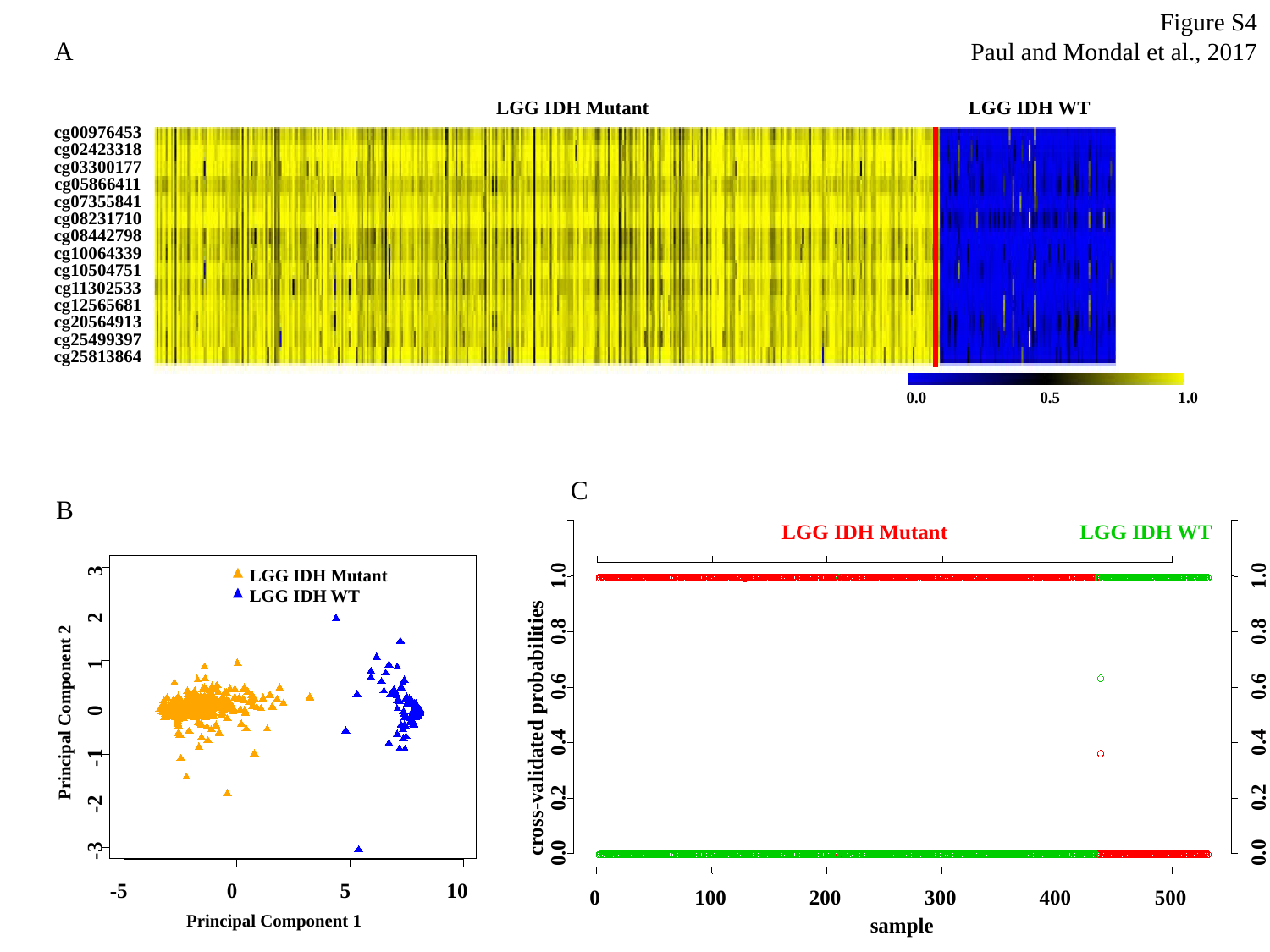

Figure S4
Paul and Mondal et al., 2017
A
LGG IDH Mutant
LGG IDH WT
cg00976453
cg02423318
cg03300177
cg05866411
cg07355841
cg08231710
cg08442798
cg10064339
cg10504751
cg11302533
cg12565681
cg20564913
cg25499397
cg25813864
0.0
0.5
1.0
C
B
LGG IDH Mutant
LGG IDH WT
1.0
1.0
0.8
0.8
0.6
0.6
cross-validated probabilities
0.4
0.4
0.2
0.2
0.0
0.0
0
100
200
300
400
500
sample
LGG IDH Mutant
3
LGG IDH WT
2
1
0
-1
-2
-3
-5
0
5
10
Principal Component 2
Principal Component 1

## Slide 6
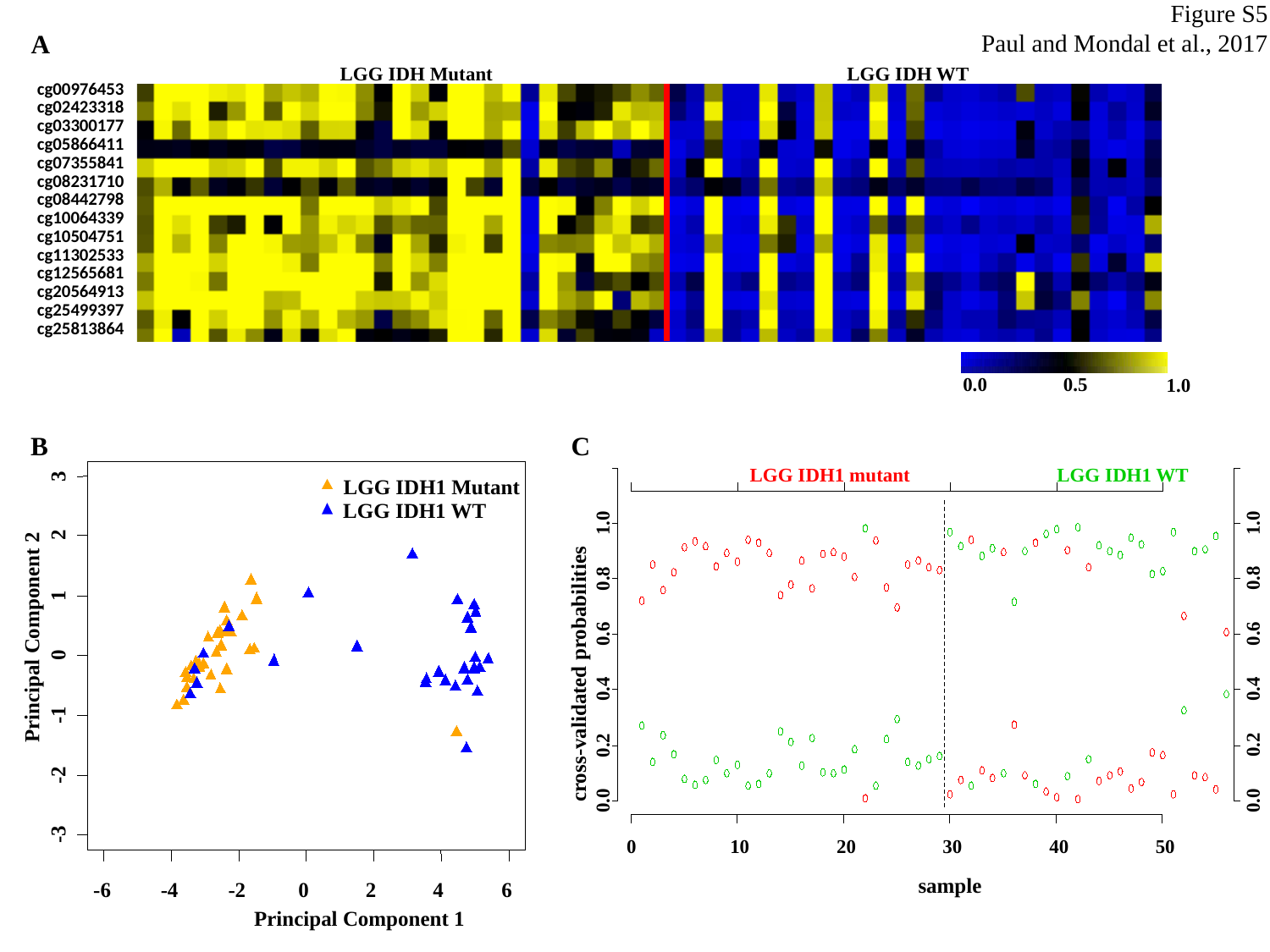

Figure S5
Paul and Mondal et al., 2017
A
LGG IDH Mutant
LGG IDH WT
cg00976453
cg02423318
cg03300177
cg05866411
cg07355841
cg08231710
cg08442798
cg10064339
cg10504751
cg11302533
cg12565681
cg20564913
cg25499397
cg25813864
0.0
0.5
1.0
B
C
3
LGG IDH1 Mutant
LGG IDH1 WT
2
1
0
-1
-2
-3
-6
-4
-2
0
2
4
6
Principal Component 2
Principal Component 1
LGG IDH1 mutant
LGG IDH1 WT
1.0
1.0
0.8
0.8
0.6
0.6
cross-validated probabilities
0.4
0.4
0.2
0.2
0.0
0.0
0
10
20
30
40
50
sample

## Slide 7
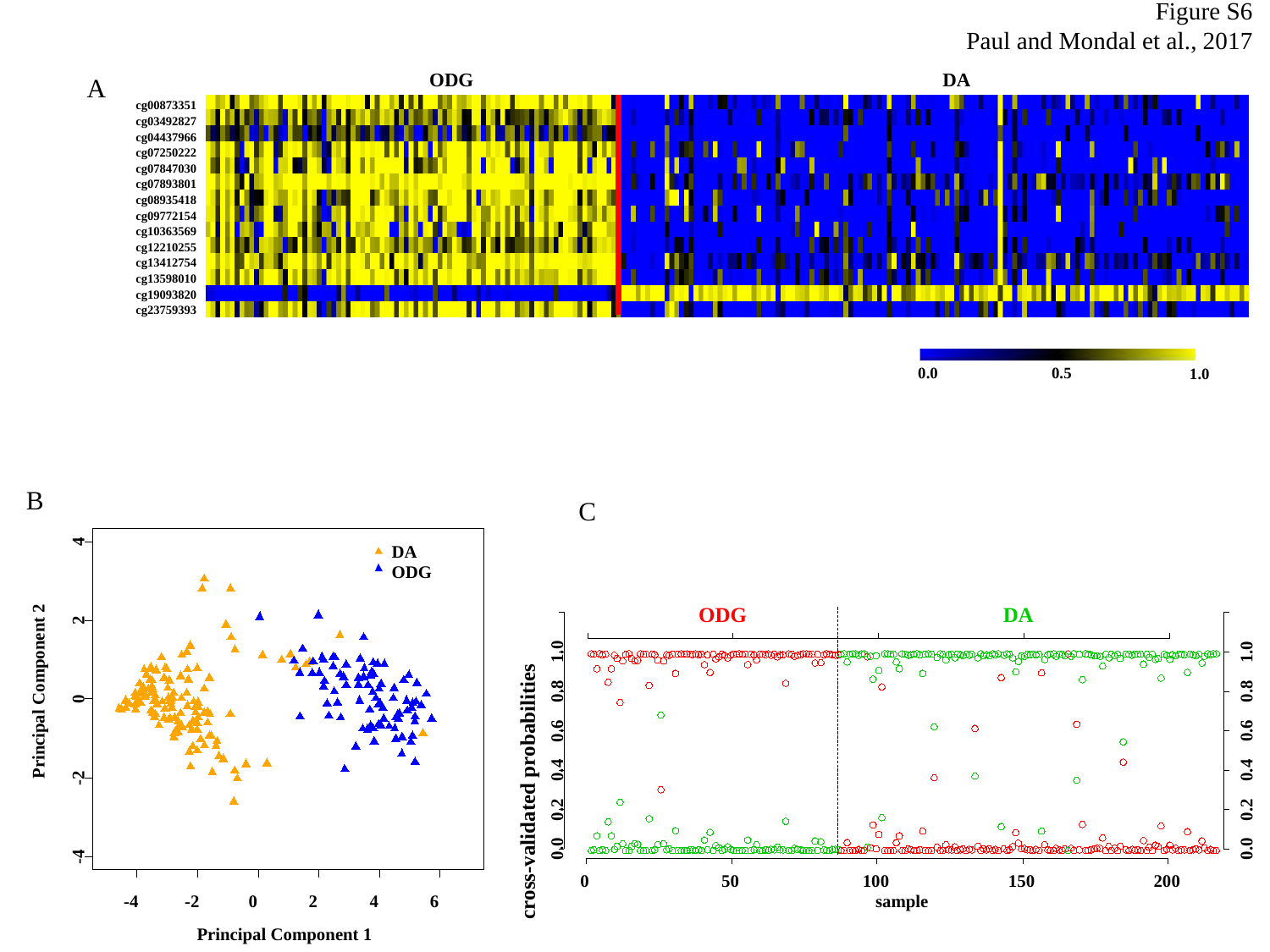

Figure S6
Paul and Mondal et al., 2017
A
ODG
DA
cg00873351
cg03492827
cg04437966
cg07250222
cg07847030
cg07893801
cg08935418
cg09772154
cg10363569
cg12210255
cg13412754
cg13598010
cg19093820
cg23759393
0.0
0.5
1.0
B
C
4
DA
ODG
2
0
-2
-4
-4
-2
0
2
4
6
ODG
DA
1.0
1.0
0.8
0.8
0.6
0.6
0.4
0.4
cross-validated probabilities
0.2
0.2
0.0
0.0
0
50
100
150
200
sample
Principal Component 2
Principal Component 1

## Slide 8
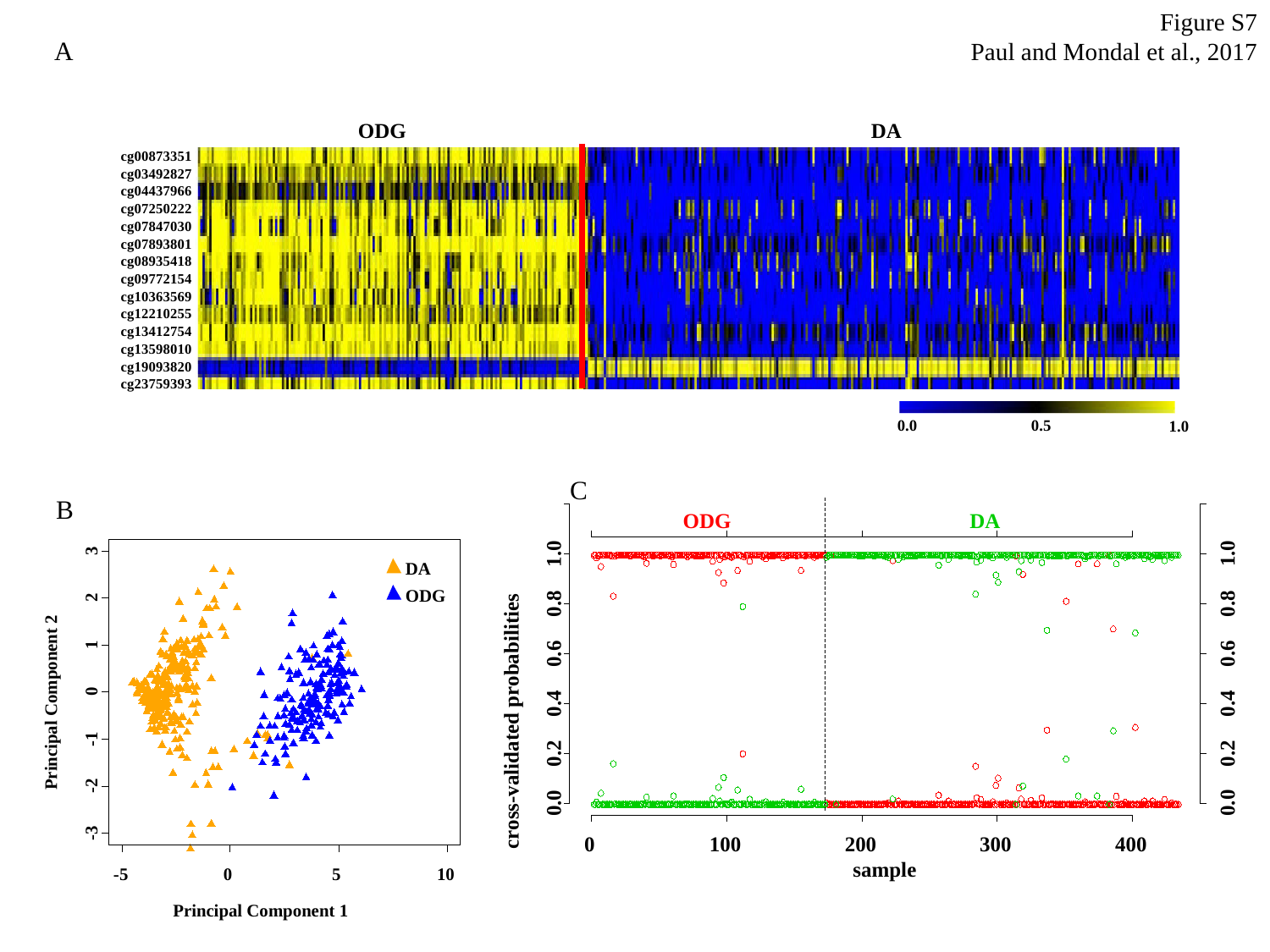

Figure S7
Paul and Mondal et al., 2017
A
ODG
DA
cg00873351
cg03492827
cg04437966
cg07250222
cg07847030
cg07893801
cg08935418
cg09772154
cg10363569
cg12210255
cg13412754
cg13598010
cg19093820
cg23759393
0.0
0.5
1.0
C
B
ODG
DA
1.0
1.0
0.8
0.8
0.6
0.6
0.4
0.4
cross-validated probabilities
0.2
0.2
0.0
0.0
0
100
200
300
400
sample
3
DA
ODG
2
1
0
-1
-2
-3
-5
0
5
10
Principal Component 2
Principal Component 1

## Slide 9
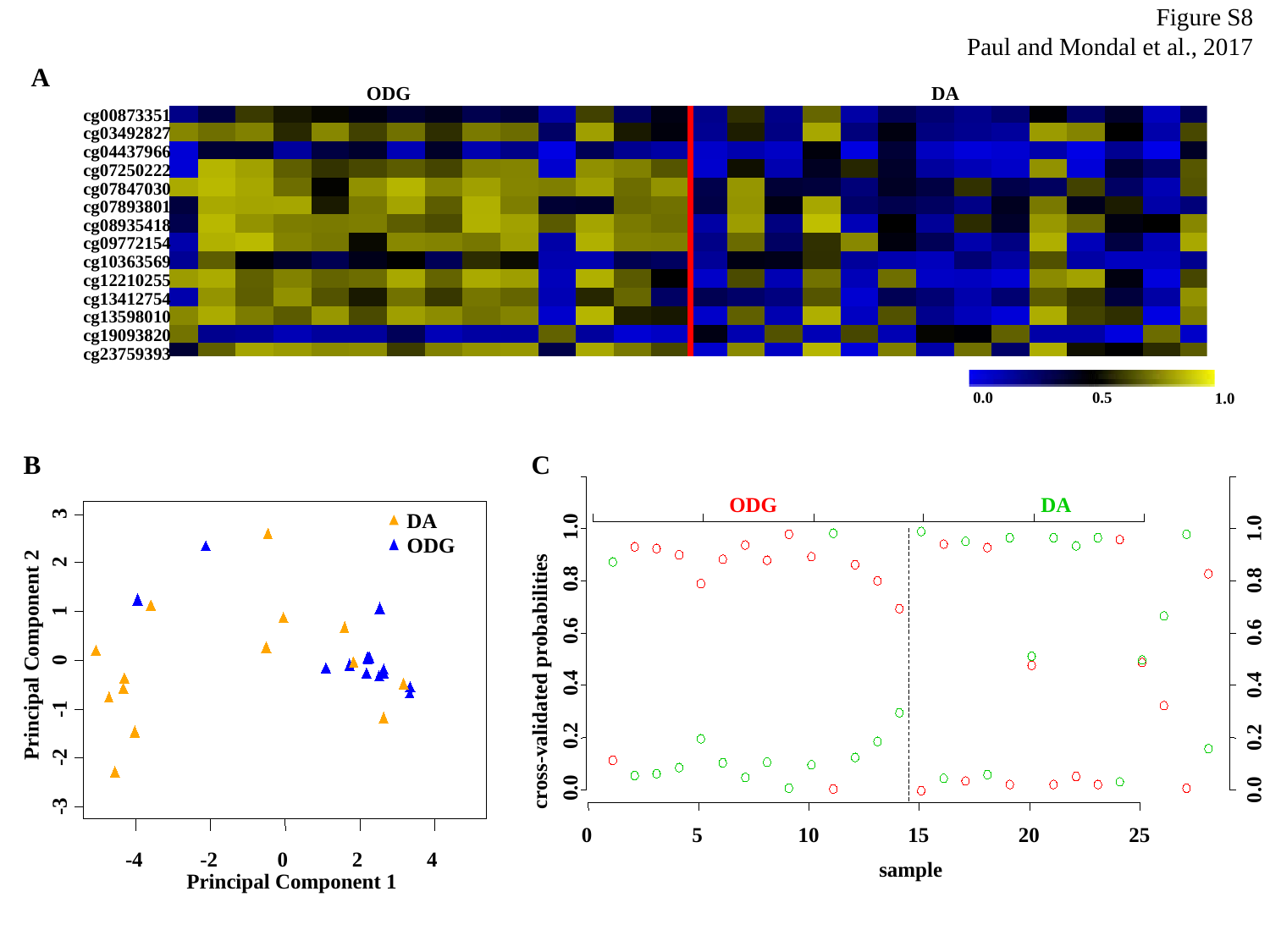

Figure S8
Paul and Mondal et al., 2017
A
ODG
DA
cg00873351
cg03492827
cg04437966
cg07250222
cg07847030
cg07893801
cg08935418
cg09772154
cg10363569
cg12210255
cg13412754
cg13598010
cg19093820
cg23759393
0.0
0.5
1.0
B
C
ODG
DA
1.0
1.0
0.8
0.8
0.6
0.6
cross-validated probabilities
0.4
0.4
0.2
0.2
0.0
0.0
0
5
10
15
20
25
sample
3
DA
ODG
2
1
0
-1
-2
-3
-4
-2
0
2
4
Principal Component 2
Principal Component 1

## Slide 10
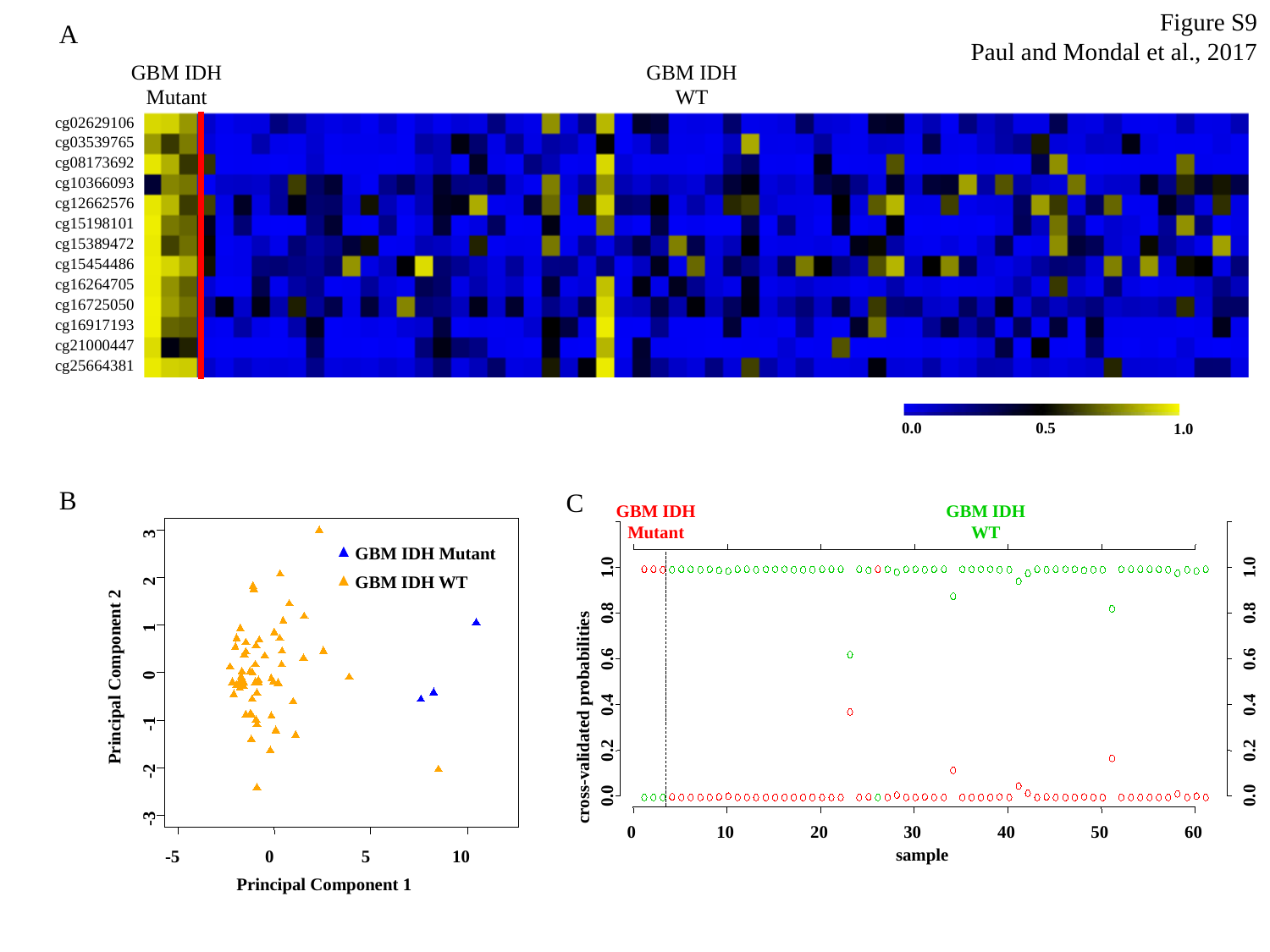

Figure S9
Paul and Mondal et al., 2017
A
GBM IDH Mutant
GBM IDH WT
cg02629106
cg03539765
cg08173692
cg10366093
cg12662576
cg15198101
cg15389472
cg15454486
cg16264705
cg16725050
cg16917193
cg21000447
cg25664381
0.0
0.5
1.0
B
C
GBM IDH Mutant
GBM IDH WT
3
GBM IDH Mutant
GBM IDH WT
2
1
0
-1
-2
-3
-5
0
5
10
1.0
1.0
0.8
0.8
0.6
0.6
0.4
0.4
cross-validated probabilities
0.2
0.2
0.0
0.0
0
10
20
30
40
50
60
sample
Principal Component 2
Principal Component 1

## Slide 11
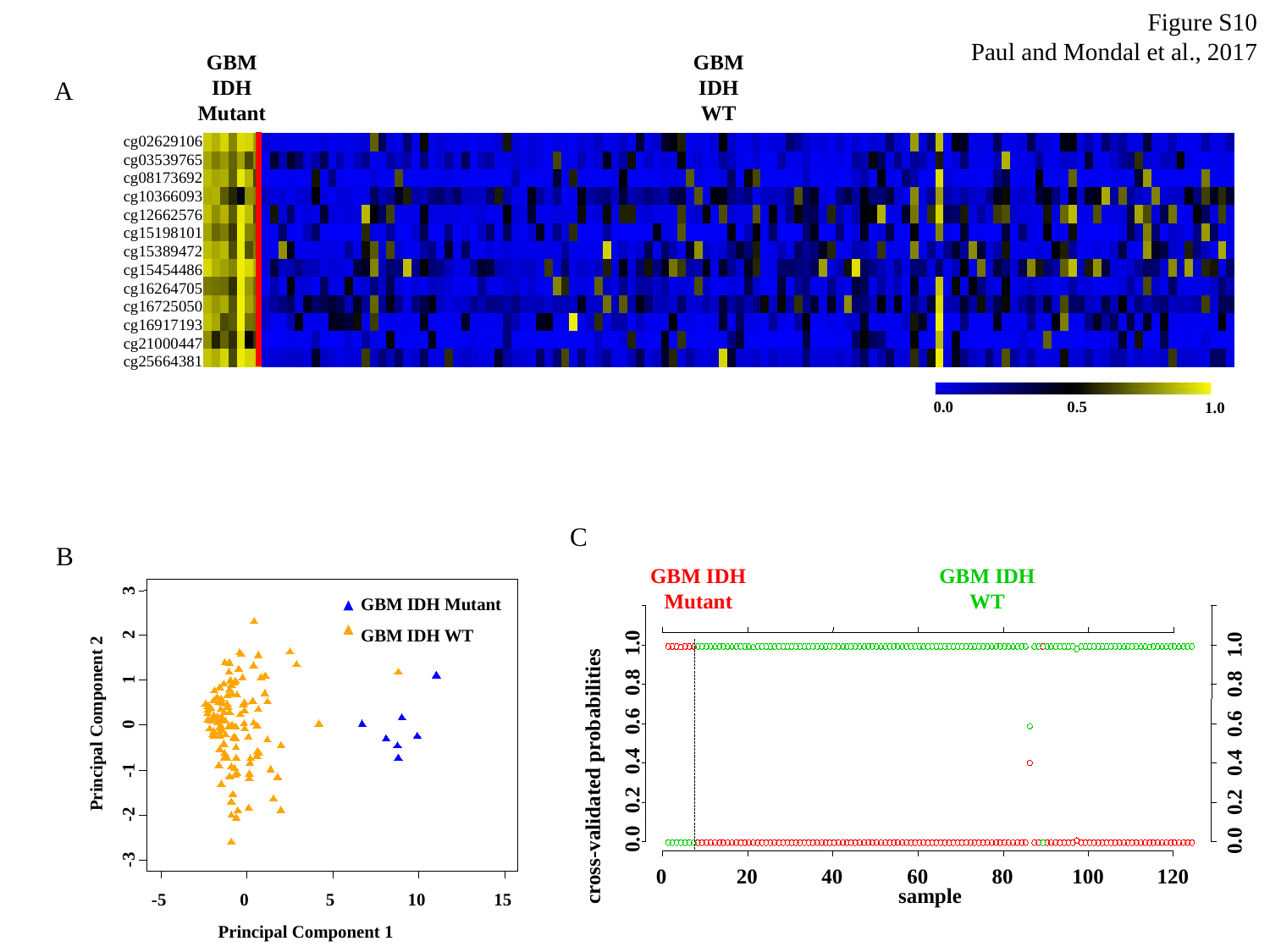

Figure S10
Paul and Mondal et al., 2017
GBM IDH Mutant
GBM IDH WT
A
cg02629106
cg03539765
cg08173692
cg10366093
cg12662576
cg15198101
cg15389472
cg15454486
cg16264705
cg16725050
cg16917193
cg21000447
cg25664381
0.0
0.5
1.0
C
B
GBM IDH Mutant
GBM IDH WT
1.0
1.0
0.8
0.8
0.6
0.6
0.4
0.4
cross-validated probabilities
0.2
0.2
0.0
0.0
0
20
40
60
80
100
120
sample
3
GBM IDH Mutant
GBM IDH WT
2
1
0
-1
-2
-3
-5
0
5
10
15
Principal Component 2
Principal Component 1
